# Good Attitudes Are Not Good Enough: An Ethnographical Approach to Investigate Attitude-Behavior Inconsistencies in Sustainable Choice

**DOI:** 10.3390/foods10061317

**Published:** 2021-06-08

**Authors:** Kathrin Barbara Meyer, Johannes Simons

**Affiliations:** Department of Agricultural and Food Market Research, Institute for Food and Resource Economics, University of Bonn, Nussallee 21, 53115 Bonn, Germany; johannes.simons@ilr.uni-bonn.de

**Keywords:** sustainability, food choice, attitude-behavior gap, household, qualitative

## Abstract

This research explores reasons for the attitude-behavior gap of consumers involved with sustainable food choice. For this purpose, the Food Choice Process Model by was applied. The study follows a qualitative approach. Data were collected through ethnographical fieldwork. Over the course of nine months, researchers repeatedly accompanied six families. Each visit lasted several hours and included multiple in-depth discussions, food shopping observations and participation in everyday food behavior. Findings show that beliefs, positive attitudes, and behavioral intentions do play an important role for sustainable choice. Rooted in one’s personal life course experiences and the socio-cultural conditions one grew up in, however, their determinacy is heavily impaired by household realities and by various personal and situational factors. Sustainability attributes, even if dominant on an abstract level, tend to be inferior for actual choice, especially when competing with the taste, price, and preferences of other household members. Product evaluation and food choice are seldomly a result of comprehensive information processing, but rather based on simplifications and strategies. Conflicts are aggravated by competing sustainability values and attributes. Confronted with diverse product-related, personal, external, and situational influences, sustainable choices come with conflicts, tensions, and ambivalences forcing participants to make compromises and remain flexible in their decisions. However, participants were aware of their inadequacies and accept personal inconsistencies, without showing much dissonance. This research extends current knowledge about the impact and the origin of attitudes towards and barriers for sustainable food choice behavior that help to understand the complexity of the phenomena in its natural setting. It points out practical implications for practitioners, updates the theoretical framework, and can widen researchers’ perspective on sustainable food choice behavior.

## 1. Introduction

The impact of food choice on personal health and the environment is a central issue in political and social debates, food-related policies, and research. Today’s food system contributes to some 20–30% of global greenhouse gas emissions, accounts for over 70% of all human water use, and is a major source of water pollution [1,2]. Population growth and economic prosperity will further increase the demand for resources and aggravates the need for their sustainable usage [3]. The Food and Agriculture Organization (FAO) (2018) [4] defines food systems as sustainable if they deliver food security and nutrition in such a way that the economic, social, and environmental bases to generate food security for future generations are not compromised. Apart from producers and other stakeholders on the supply chain, households do influence the sustainability of food systems through their consumption patterns. Previous research shows that consumers associate sustainable consumption with eating less meat and animal-based products [5], producing less food waste [6] and packing waste [7], and consuming organic food [8,9] as well as local food [10]. In Germany, public surveys postulate that there is in general a positive attitude towards sustainable consumption [11]. Over 90% of German consumers state that protecting the environment is important to them and acknowledge their role with respect to that [12]; 62% say that the origin of the product is one of the most important purchase factors, and 35% declare that the food’s compliance with ethical beliefs is one of the most crucial purchase factors [13]. Academic literature supports the existence of positive attitudes toward sustainable food products. Organic and local foods, for example, are associated with safety, better taste as well as social, health, and environmental benefits [14,15,16,17]. However, the markets do not reflect those positive attitudes and perceptions. The share of organic food in the German food market remains low with only around 5.7% in 2019 [18]. According to a survey amongst producers and processors of local food, distribution takes place via various sales channels depending on the product group and producer size. Although 60% sell their products via retailers, the main distribution channels remain farmer markets and farmer shops [19], while German consumers mainly visit supermarkets and discounters [20]. Finally, meat consumption in Germany only slightly declined in recent years but remained relatively constant over the last two decades with about 60 kg per capita [21]. Thus, there is evidence for the so-called attitude behavior gap which implies that despite consumers having a positive attitude or even buying intentions, the actual purchase fails to appear [22,23,24,25,26,27,28,29,30,31,32,33,34].

Past research reveals that intrapersonal factors, such as conflicting goals [35], a lack of trust [36,37,38,39], a lack of knowledge [40,41,42], and a low willingness to pay [38], and contextual factors, such as higher prices [34,40], a low availability of sustainable products [9,34], and a perceived lower quality [34,43,44], are some of the main reasons for the attitude-behavior gap for green products (For a comprehensive overview of the studies investigating the green gap including methodologies and paradigms used, we recommend reading the recent literature review by ElHaffar, Durif, and Dubé (2020) [29]). In their recent literature review on studies investigating the attitude-intention-behavior gap, however, ElHaffar, Durif, and Dubé (2020) [29] indicated that more qualitative studies and experimental designs are needed to investigate the phenomenon. The majority of the existing literature—they argue—applies economic rational paradigms to investigate the phenomena, mainly the Theory of Planned Behavior [45]. The central assumption here is that individual attitudes positively influence behavioral intentions and ultimately behavior. By addressing consumers as rational decision-makers that are somewhat disconnected from wider sociocultural processes [46,47], however, rational and behavioral paradigms ignore that individuals do not live in a social vacuum and that food choice is an elusive, multi determinant, context-dependent phenomenon [48,49]. Constructionist research approaches try to move beyond individualist rationalistic thinking and enrich our understanding of sustainable consumption by conceptualizing consumption as a social and cultural practice that is less rational and less oriented toward individual needs [46,50]. In this study, we further investigate sustainable food choice behavior and evident inconsistencies through an ethnographic approach with families who define themselves as “sustainable consumers”. By using the Food Choice Process Model (FCPM) by Furst et al. (1996) [51] as a theoretical framework this research investigates sustainable consumption from a holistic perspective and through its forms of practice. This study aims to investigate (1) how attitudes towards sustainable food form over the life course and (2) how personal, external, and situational influences affect (sustainable) food evaluations and choice. Furthermore, the paper aims to unravel (3) the mental process employed in everyday life when shopping for (sustainable) food. Finally, the study tries to gather (4) insights about how pro-sustainable consumers deal with inconsistencies.

## 2. Theoretical Framework

The FCPM [51] was chosen as a theoretical frame because it considers food choice as a process involving physiological, cognitive, and sociocultural influences and processes [51] influenced by a multitude of factors at different levels. In the past, the model and components of it have been used to investigate the food choice process of the elderly [52,53], life course influences on fruit and vegetable consumption [54], women’s dietary prevention motives [55], how people manage healthy eating behaviors [48], peoples’ food classifications [49], food choice capacities [56] and eating episodes, and food scripts and routines [57]. However, to our knowledge, the FCPM has never been used to investigate sustainable food choices or the attitude-behavior gap.

**Figure 1:**
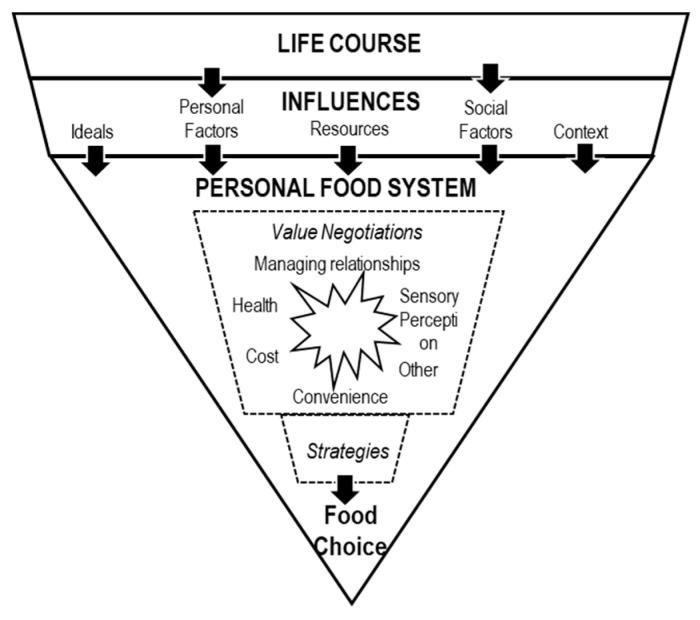
The food choice process model adapted from Furst et al. (1996) [51] and Connors et al. (2001) [60].

The model consists of three main components: the life course, the influences, and the personal system. The life course reflects one’s individual food choice development within the micro- and macro context over time [58]. It includes trajectories and transitions and turning points. Food choice trajectories incorporate “a person’s persistent thoughts, feelings and strategies and actions as she/he approaches food choice” [54]. They are relatively stable over an adult life although they have some transitions and a few turning points, caused by changes in a person’s life [54,59]. Influences refer to ideals, personal factors, resources, social factors, and the food context. Ideals provide reference points for individuals to evaluate and judge food behavior. They are expressed when people describe something as “the right”, “the normal”, or “the bad” way to behave. Personal factors include individual predispositions and food preferences or aversions, as well as self-attributed roles [51,58]. Resources are (un)available assets for food choices. They include financial resources but also material, human, social, and cultural resources. Social factors refer to the system of relationships an individual is embedded in [51,58]. The food context encompassed the physical surroundings and specific food supply factors in the environment such as food sources and availability of foods in the food system [51]. A person’s ‘position’ in life, the first component of the FCPM, determines the influences and thus the second component of the conceptual model, e.g., being a student or being a full-time working graduate influences the money one can spend on food. The third component, and the core of the model, is the personal food system. It refers to the mental process used to decide on a specific food. The first element of the personal food system refers to the value negotiation. Hereby, various food values like taste, convenience, and costs are consciously “contrasted with each other and judged according to their significance in a specific food choice” (Furst et al. (1996, p. 257) [51]. The second element of the personal food system refers to the usage of strategies that guide food choices. Figure 1 shows the Food Choice Process Model adapted from Furst et al. (1996) [51] and Connors et al. (2001) [60].

## 3. Materials and Methods

### 3.1. Sample and Data Collection

To understand how phenomena fit into the complexity of people’s lives, ethnographical approaches use in-depth-discussions combined with observational methods to study behavior in its natural setting. The advantage of studies using ethnography is that they go beyond accessing what people say they do and investigate what they really do in practice. For gaining insights into the symbolism and meanings of behavior, researchers must be present in the field and participate in social life [61]. Ethnographical research is time consuming as it relies on extensive fieldwork. Besides data from observations and formal interviews, informal interviews and casual conversations taking place for instance while eating, drinking coffee, or cleaning the kitchen are an essential resource of “experience near” information in ethnography [62]. Thereby, ethnography offers the potential to provide “rich data” and develop a “thick description” of social behavior even though it often only uses small samples.

Within this ethnographic study, the sample consisted of six households from North-Rhine Westphalia, Germany. As the aim of the study was to gain insights into the attitude-behavior gap, all included households were involved with healthy and pro-environmental food behavior. Families were recruited over social media postings and flyers. Ten families applied for the study. Involvement was determined using the families’ application emails, in which they have been requested to elaborate on their family setting and their general food behavior, e.g., “How many people are living within your household?”, “How many of those people are under the age of ten?”, “What is important for you when shopping for food?”, “Is there something in particular you pay attention to? “The six families selected indicated a positive attitude towards sustainable food consumption, e.g., paying attention to organic, local, and seasonal food. The study focused on families with diverse cultural backgrounds and with children at different ages, as the literature suggests that interest in sustainable food choice increases within households with babies, decreases as children become older, and increases again when the children have left the household [63]. The study did not include young single households as financial restraints usually prevent them from shopping higher-priced products [16,64]. Pre-meetings were conducted to confirm households’ self-attributed involvement and to inform them about the course of the study. Over nine months in 2017/2018, each of the six families was visited three times: once during summer, once during fall, and once during the winter season. Each visit lasted four to six hours. Data were collected using in-depth interviews; dialogic conversations; photographs send by the participants; and observations (audio and video recorded) of and participation in the planning of grocery shopping, grocery shopping itself, food preparation, cooking, and eating (having lunch/dinner together). Researchers did not take an active role in the planning of grocery shopping. Most participants decided what they were going to prepare for dinner/lunch before the researchers arrived. Whilst shopping, participants bought the ingredients needed for lunch/dinner and additional foods needed in the household; researchers observed the shopping trip and occasionally asked why a specific product was chosen.

During each visit, there were semi-structured interviews and in-depth conversations. The discussion structure was quite flexible, allowing the researcher certain freedom in asking questions or following up a discussion. On the first visit, the researchers brought a basket with food items with sustainability attributes (local, organic), some carrying (different) food labels. This facilitated an extensive discussion about food quality schemes. Dialogic conversations with participants were introduced on the second and last visits. The purpose was to ask questions, observe, and discuss the filmed, photographed, and noted practices together. This self-reflexive approach sought to better understand practices through common reflection and discussion. To stimulate the dialogic conversation, on the last visit, cards representing labels were used as a concrete basis for discussion. During all visits, researchers had the opportunity to inspect households’ fridges and larders, thereby gaining insights into participants’ grocery stocks. Between the visits, the families were supposed to send photographs documenting their everyday consumption habits (e.g., for the first visit: a typical breakfast during weekdays and holidays, for the second visit: documentation of the food eaten over the course of a day). Those photographs were also used in dialogic conversations at the subsequent visit. During each visit, two researchers were present. In total, three researchers were involved in the study. To assure consistency, the first author of this article accompanied each visit, to establish mutual trust between the scientists and the families; the same two researchers attended all visits to one specific household. Each household received an iPad as an incentive for taking part in the study. Table 1 shows the main characteristics of the households.

In-depth interviews and food preparations were video recorded. Shopping tours were audio recorded, as German retailers do not allow video recording in stores. Recorded data resulted in over 400 written pages of transcript and over 100 photographs sent by the participants. Examples of the data recorded by researchers and received by the participants throughout the study can be found in the Supplementary Material SA.

### 3.2. Data Analysis

Data collection and analysis was an ongoing and integrated process during the study. NVivo (Version 11.4. QSR International) was used to manage and analyze data. Based on a hybrid approach, we used deductive and inductive research techniques. Through the inductive approach, topics that were important to understand food choice behavior were identified. An example of an inductive code is “DIY” with the main theme “self-made dishes are associated with health benefits”. In the deductive approach, the FCPM served as the theoretical framework. An example is the code “cost” with the main theme: “Though prices of a single sustainable product are rejected as competing value, participants admit that exclusively shopping for sustainable products would be too expensive”. Initially, the first author coded the transcripts. After the first inductive codes and codes deduced from the FCPM were established, the author retrieved the quotations, created connections between the codes, and recoded the data. The development of codes and themes was an iterative and reflexive process between both the authors. With new themes and dimensions arising from data analysis, the Food Choice Process Model was modified and extended concerning sustainability aspects. Based on constant comparison [65,66], data from new visits were compared with data from former cases leading to a continuous process of revising codes and categories until theoretical saturation was achieved. The codebook, including main themes, is attached as Supplementary Material SB. It is important to note that while we used theoretical propositions from the FCPM to conceptualize the data, these were not explicitly presented in the research questions. They emerged through the process of interpretation, analysis, and discussion among the researchers.

## 4. Findings

### 4.1. The Life Course

Findings show that the macro context, e.g., the socio-historical and economic environment participants grow up in, forms food trajectories. These develop over the life course and affect consumption practices and thus the awareness and importance one attaches to sustainability of food-related issues as an adult. For example, Margot, born in the early 50s and living within a family that was struggling with financial issues back then said: “*When I was a child, homegrown food was the only thing we had* *and this is where my positive attitude*” *towards local and seasonal food* “*comes from” [Margot]*.

Additionally, participants’ cultural identity did influence food choices: For instance, Selda, whose parents grew up in Turkey, said that she “*loves*” to shop at the Turkish supermarket and that this is her “*passion*”. Even though she normally pays attention to sustainability characteristics like local or organic, here she only listens to her “*Turkish heart*” and just takes the things “*she is used to*” from childhood. Both Adil and Cem refrain from eating pork, however, not because of religious reasons, but because they are not accustomed to it. Furthermore, meso- and microlevel factors such as family traditions and childhood habits influenced participants’ food trajectories. Nina, for instance, explained her preference for shopping from retailers’ counters instead of off the shelf as follows: “*Back then we always went grocery shopping on Fridays and my parents brought fresh cold cuts from the butcher, we sat together … it was a ritual” [Nina]*. Jennifer complained that her partner Cem occasionally buys an excessive number of specific products (e.g., sweetened fruit yogurt or bread loaves) just because they are on sale, although the family is hardly able to consume all of it. He justified this as being a vestige from his upbringing, as his parents always wanted to make sure that “*all mouths are fed*”.

Although most habits of consumption are formed in early childhood, they are not static and can change in sensitive periods of life. For Laura, Jennifer, and Julia, pregnancy and childbirth represented turning points that were capable of raising the importance they attach to sustainability issues: “*I just got into the issue [of sustainable food] when [her son] got into nursery. During my studies, I did not care at all” [Julia]*. For Margot, an early illness of her husband Holger was a turning point: “*During rehab, they gave talks about healthy diets, and that motivated me and I thought ’ok, that’s what we need to pay attention to from now on’” [Margot]*. In Seldas’s case, a recent divorce shifted the family’s diet further away from being sustainable, as financial constraints prevented her to shop at the organic supermarket frequently. Entering working life (e.g., from previously being a student), a new job, or family relocations also lead to shifts in the trajectories. However, the impact of such incidents seems to be less substantial: “*During my studies that was a highlight, buying such a thing [frozen lasagna] […] going to let myself go and bum around*” *[Jennifer]* “*Frank ate a lot of meat before he moved here. […] it stopped when he moved here […] But well, he still eats it for every lunch break at the canteen*” *[Julia]*.

**Figure 2:**
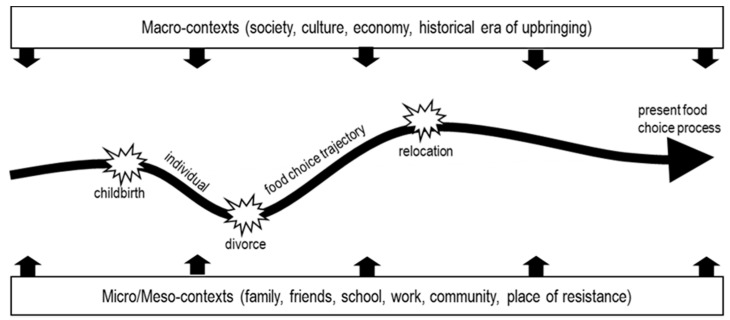
A conceptual model of the life course perspective on sustainable food choice. Adapted from Devine et al. (1998) [54]; Sobal et al. (2006) [58].

Figure 2 illustrates decisive life course events that affected participants’ food choice trajectories with respect to sustainability.

### 4.2. The Influences

At the next stage, the framework highlights the influences on food choice. Unsurprisingly, all participants showed a positive attitude towards sustainable consumption. This attitude was strongly influenced by personal **ideals** and expectations about “how one should behave”. Ideals concerning the sustainability dimension health were rather self- and family interested, including self-prescribed rules used to ensure a healthy diet for the family: “*The rules here are: as little sugar as possible, as little flour as possible and as many fruits and vegetables as possible*” *[Jennifer]*.

Ideals that are rooted in concerns about the role of the food sector on environmental pollution and climate change, however, were driven by more altruistic motives: “*That the food has been produced in a good structure, that its cultivation is environmentally friendly and climate and animal friendly […]. Sustainability is that we do not mess something up for the following generation due to our consumption*” *[Julia]*. They were accompanied by concerns about one’s own impact on environmental and societal problems and a desire of acting morally good. Jennifer for example was convinced that it is “*somehow her duty*” to pay attention to Fair Trade when shopping for coffee and that society is obliged to change consumption patterns in order to secure the wellbeing of following generations: “*We are the last generation that is able to change things*” *[Jennifer]*.

However, concerning **personal factors**, especially personal preferences were rather hard to overcome, when it came to translating positive attitudes into behavior. Selda, for example, had a negative attitude towards Dutch tomatoes and refused to buy them, regardless of whether they were sustainable or not. Interestingly, she was not able to explain the basis for her negative bias. The determinacy of personal preferences became also obvious in the case of imported fruits. Even though all participants criticized long transport distances and high-water usage during cultivation, all participants (except for Margot) stated that they buy some fruits important to them regularly: “*Avocados, I buy them sometimes if I crave them*” *[Jennifer]*. “*Well, I do buy products, mango or kaki, which are not from here. I don’t want to be restricted here” [Nina]*. “*We love pomegranates and we just buy them, no matter where they come from*” *[Laura]*.

Taking over food roles for example “being responsible for the family’s diet” or identifying oneself as a vegetarian/vegan reinforced sustainable consumption practices. Although Laura, Julia, and Jennifer admitted that it was too “*hard*” to implement a vegan diet in the long run, Julia managed to follow a vegetarian diet for several years, and both Laura and Jennifer were able to implement a vegan diet for short periods in the past. Another interesting finding is that food provisioning still followed rather traditional family roles: Though, the whole family was invited to our discussions, during more than 60% of all visits, only the female adult was present. If both adults were present, females were much more involved in the discussions generally, and specifically in sustainability issues. Moreover, it seemed that within their role as the “caretaker”, they felt personal and sometimes solely (Laura, Margot, Jennifer) responsible not only for grocery shopping but also for ensuring a healthy and sustainable diet for their family: “*If I send Adil then I have to write him a list, but I tend to do it myself or give him precise instructions” [Laura]*. Although they play this role voluntary and deliberately, it puts a lot of pressure on them: “*Of course, I do the cooking most of the time. I have a child and I want it to grow up as healthy as possible. Sometimes I hate that role, but it is because of the child*” *[Jennifer]*.

The availability of tangible and intangible **resources** can either limit or support the implementation of a sustainable diet. All participants admitted that there is no time for extensive information research. Instead, their knowledge about sustainable food products came from narratives from friends and family and fragments of (social) media information: “*There is always something on Facebook:* ‘*You should not buy those things: Nestlé, Danone…*’” *[Jennifer]*. “*There is one Facebook page called* ‘*sustainable living*’ *a friend linked me there*.” *[Selda]* which can be hard to remember: “*I once read a study, a Swedish one, with people who only ate organic and people who did not, and their health values were measured*—*I don’t know which ones. And the ones who ate organic were healthier*—*regarding those basic values*” *[Jennifer]*. This knowledge was easily compromised by contradicting messages and a lack of faith in the reliability of the information, which lead to ambivalences and uncertainty: “*Yes, I think it [organic food] is better. But I am not very involved with this issue. Lately, I heard that it [organic] is not a protected term*” *[Adil]*. “*There are different organic symbols in the organic section. And one roughly knows that Demeter* (Demeter is the largest certification organization for biodynamic agriculture. It is a popular organic Food Label Scheme in Germany. It is perceived to be the strictest certification in terms of environmental friendliness and animal welfare by German consumers [67] *is the badass. But that’s all too unclear for me or maybe I did not follow up closely enough with that […] as for now, it disorientates me*” *[Julia]*.

Providing participants with more information did not inevitably affect choice either. During the first visit, researchers explained and discussed different Food Quality Schemes in detail, although all interviewees were interested and showed a positive attitude towards most labels, the information provided did not have a persistent effect on resolving uncertainties, nor did it seem to affect their shopping behavior decisively: “*I did not pay more attention to that, I shopped like I did before*” *[Julia]*. One participant even forgot about one of the previously discussed labels completely.

Participants’ **financial resources** did play an essential role in food choice. All participants indicated that a comfortable and safe financial situation is a precondition for sustainable consumption: “*It might be that it depends on my income which is rising because I am not a student anymore. I think the more you can spend, the better you live. Someone on social welfare will not be able to buy organic, that’s obvious. I can see progress for me, the better it gets with the job, the more attention I pay on organic and quality food” [Julia]*. Adil who is a student said: “*I can’t afford organic at this point anyway*” after being asked why he did not choose the organic alternative during grocery shopping, although he thinks “*it is better*”. However, even within a financially comfortable situation, participants admitted that visiting organic shops regularly would exceed their financial capabilities: “*I could never shop at the organic supermarket for a whole month*” *[Julia]*. “*If we had the money we would exclusively buy organic meat, in this case, I would shop at the organic butcher, however, most of the time we cannot afford that*” *[Nina]*.

According to Margot and Selda, cooking skills are another important resource supporting sustainable food practices. However, cooking skills were mentioned simulations with the respective equipment, which then again comes back to sufficient financial resources: “*I have good equipment, I do have a steamer, I have a very good oven and I can cook*” *[Selda]*.

Besides financial, material, and human resources, participants mentioned that the **availability of time** affects their ability to implement a sustainable diet. Because females feel responsible for providing a sustainable diet for their families, they are in a constant struggle to balance work, family, and other responsibilities. Concerning the **food context**, participants prefer to visit alternative retailers like organic supermarkets, small-scale sellers, and farmer markets which were strongly associated with sustainable consumption. However, the implementation of sustainable practices requires additional time: “*It is hard if I am at work all day and also have appointments in the evening, these are the worst days regarding our diet*” *[Jennifer]*.”*Recently we haven’t been there [at the framers’ market] often, because I am working, there was no time to drive there*” *[Nina]* especially if the distribution of such retailers in the immediate neighborhood is low: “*I did this in Berlin very often. I had four to five stops during a shopping trip. That was an advantage of Berlin: many organic shops, pretty close, also different ones and big organic supermarkets*” *[Laura]*.

Particularly for participants with smaller children, grocery shopping has to fit in somewhere between work (Nina), picking up the children from school/daycare (Jennifer and Julia), and other household responsibilities. Additionally, grocery shopping at alternative retailers bears the risk of a limited assortment. Margot for example had to dismiss the recipe she originally planned to prepare because asparagus was not available at the organic farmer shop we were in. Furthermore, she told us that she bought salmon at a discounter beforehand, as she was unsure if it was available at the farmer shop. Time restrictions, a low distribution of preferred retailers in the immediate neighborhood, and their sometimes-reduced assortment contradicts with participants desire to get over with grocery shopping as fast, safe and conveniently as possible: “*[I am going to that supermarket] because of a lack of time. There I have the feeling I can get everything. If there is more time, I like to visit more shops [Laura]*”. “*If I can avoid it [visiting several shops], I prefer getting things done in one shop*” *[Julia]*. Thus, they usually visit conventional supermarkets and discounters, even though they are not satisfied with the assortment in terms of sustainability. Julia, for example, did not buy organic milk while we were shopping at the discounter because the discounter did not offer organic milk without lactose.

Observations of grocery shopping and investigating the photo documentations support these findings. Although all participants complained about the amount of plastic packed food at supermarkets and stated that they try to shop for less-packaged food: “*What bothers me at [the discounter] is that the fruit and vegetables are extensively packed in plastic*” *[Jennifer]*. However, the received photos, indicate that this intention was rarely implemented. Only a few pictures showed unpacked foods, bought from farmer’s markets. The majority, however, revealed that participants buy plastic-packed food from supermarkets and discounters. In addition, besides a generally positive attitude towards organic, a lot of products bought came from conventional agriculture shops (see Supplementary Material SA).

Food choice is also heavily affected by **social factors**. Most eating takes place in the presence of others and grocery shopping incorporates food purchases for all household members and for several days. To prevent conflicts and to keep up the harmony with the family, trade-offs were unavoidable. This holds for meeting with friends: “*If I meet with friends, then I know we’ll eat a lot of sweets, drink alcohol, then I think: I don’t want this. Actually, I want to keep on going with my healthy phase*” *[Jennifer]* and as reported by Laura, Cem, and Frank (reported by Julia) for lunch at the workplace.

At home, conflicts seem to be intensified. Laura and Jennifer complain about their partners not paying attention to fruits’ country of origin, and Jennifer complains that Cem sometimes even acts (unintentionally) as a bad role model: “*Then Cem sits here with a bottle of Fanta and of course the child also wants Fanta. That’s one of those conflicts. Then I say: ‘No, that’s for grown-ups, but I think ‘why don’t you drink water and drink Fanta when the child is in bed*” *[Jennifer]*. Children’s needs and preferences seemed to be crucial for food choice. That is because children can be picky eaters and the mothers have to make sure that they eat anything, even if that is the less sustainable choice: “*[Child’s name’] only eats [a specific brand of sausages], whether I want it or not*” *[Selda]*.“*If we are at the supermarket somewhere and [name of child] wants sausages which are not organic, then I do buy non-organic*” *[Julia]*. Especially the mothers of younger children seem to be in a constant struggle trying to shape the food choice of their family members towards a sustainable diet, on the one hand, and incorporating the different preferences of their household to maintain harmony in the family, on the other hand, often sacrificing personal ideals or preferences: “*It’s just hard sometimes if you have someone [Cem] who doesn’t care at all*” *[Jennifer]*. “*Sometimes I do things because it is important for Laura. I do pay attention to some things, but that’s rather her thing […]*” *[Cem]*. Furthermore, participants with younger children (Laura, Jennifer, and Julia) try to avoid grocery shopping with their children because it is much more stressful and requires a lot more time.

### 4.3. The Personal System—Value Negotiation

According to Furst et al. (1996) [51], the life course and the influences shape the **personal system** in which food choices are made. It refers to mental processes whereby consumers translate influences upon their food choices into how and what to choose in a particular situation. Hereby, the value negotiation involves weighting different values in a food choice situation and deciding which one(s) is (are) the most important for that particular decision. In our study, participants referred not only to the five food values of the original FCPM—sensory perceptions, convenience, cost, health, managing relationships—but also to three additional ones: animal welfare, environmental protection, and social responsibility. However, sustainability and its related pillars seem to be fuzzy concepts, only accessible through certain product attributes and consumption practices: “*At least with respect to a more species appropriate animal husbandry, organic is somehow an improvement, compared to conventional animal husbandry. And this clearly is a buying incentive for me*” *[Julia]*. Selda thinks organic food is “*Just healthier for the body, and eco-friendlier. There is this environmental effect and I basically support this*” *[Selda]*. Jennifer stated that: “*Food is a big issue with all those pesticides, where does it come from and of course things as local and seasonal and so on. Fair Trade … just because I know this for coffee, that it is much better for the famers if they get support*” *[Jennifer]*. Additionally, participants reported that choosing sustainable food feels good*:* “*To me, it’s worth it, [at the organic supermarket]. I shop with good feeling*” *[Laura]*, however, that feeling did not always have a lasting effect: *“Well, I do have a better feeling if I do shop consciously […] however, this feeling is not that overwhelming that it’s present at the next shopping trip and I want to do it again” [Julia]*.

Although sustainability values are important for our interviewees, they are competing with sensory perceptions (e.g., taste, freshness), convenience, cost, and managing relationships which rather satisfying individual or family goals. Especially, taste, managing relationships, and costs seem to be crucial values often inducing conflicts. Laura for example said: “*When I don’t [buy organic] is when [buying] salami, because I don’t like it. And if I eat cheese for two weeks, I get sick of it and then I buy the normal salami*” *[Laura]*. Margot stated that the taste of the organic sausages from the farmer’s shop does not fit the traditional dish she was preparing with us, so she bought the conventional alternative. Besides taste, shelf life is an important sensory attribute. Julia and Selda, for example, complained that some organic fruits and vegetables rot faster than conventional ones and that is why she does not buy them. Laura said: “*We use a lot of milk, and if I have to buy three packages because I can’t go shopping in the next few days, I’ll buy two organics and one which has a longer shelf life*” *[Laura]*. Margot, who regularly shops and orders food from a farmer’s shop, refrains from ordering milk, eggs, and cheese there. In her opinion the delivered eggs are “*too small”*, the cheese is cut in “*stupid pieces*” that are difficult to slice, and the milk is about to reach its best before date. Sustainability values were also inferior in cases where they threatened the harmony within the participants household or endanger the nutrition of the children: “*I like whole seeds bread, if I would shop for myself I‘d take that. But they [the children] don‘t like it*” *[Laura]*. “*I do buy, what he [pointing towards her son] eats*” *[Selda]*. During our first grocery shopping trip with Laura and Adil for example, we asked Laura why she bought non-organic creme cheese. She was surprised and said she did not know that this retailer brand also offers an organic counterpart. During our last visit, she told us that she tried the organic cream cheese; however, her children rejected it, and as a consequence, she keeps on buying the conventional one.

**Figure 3:**
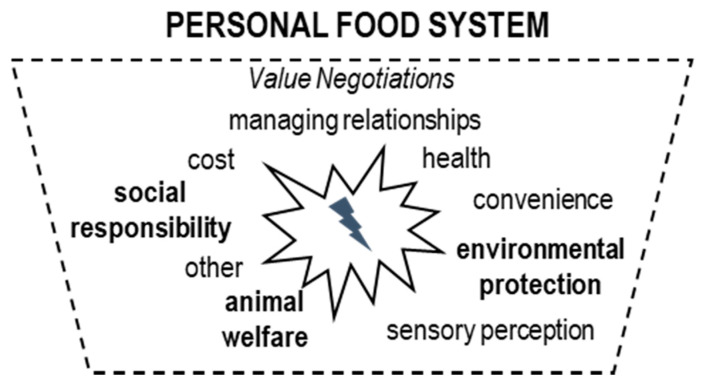
Value negotiations in the personal food system of consumers involved with sustainability.

Although the cost of a single product is hardly ever mentioned as a barrier in the discussions, our observations showed that product prices do play an important role during food shopping and that all participants examine product prices at the point of sale (PoS) (except for Julia whilst shopping at a discounter). It seemed the cost of one single product was not what impeded sustainable choice but rather the fact that participants needed to balance the accumulation of food costs. Thus, when shopping for larger quantities of foods, costs can outdo pro-sustainable attitudes. Margot for example, who usually purchases organic fruits and vegetables, uses conventional strawberries when cooking marmalade because prices are three times higher than for conventional strawberries, and she needs several pounds. Figure 3 shows the value negotiation of consumers involved with sustainability.

### 4.4. The Personal System—Strategies

This component of the FCPM acknowledges that many food decisions are less mindful and rather automatic, as they are usually routine and reoccur frequently. Consumers use strategies and simplifications to minimize their cognitive effort. While each individual has his/her own strategies and those are unique for different situations, they are generally based on similar patterns and tend to be stable [51,60].

Our participants used a variety of product attributes to assess the degree of sustainability of food products. Organic food was associated with higher animal welfare standards and with higher environmental standards and due to the absence of agrochemicals it was also perceived as being healthier: “*I’d think, someone that only eats organic food, lives healthier […] I would also go that far to say through this cultivation, soils and the land are less stressed and contaminated; that again benefits the nature, let’s say insects, birds and everything following that […]it’s probably better for all of us*” *[Selda]*. Local food was perceived as more environmentally friendly as well, mainly because of shorter transport distances. Furthermore, it is assumed to be beneficial for the local economy: “*Because it is fresher because I think I’m supporting the region […]*” *[Julia]*.

**Figure 4:**
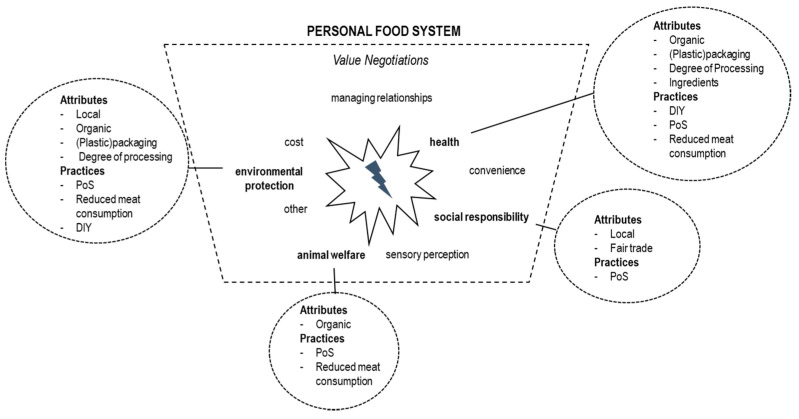
Attributes and practices participants associated with sustainability values.

Besides those specific attributes, sustainable consumption was associated with certain consumption and purchase practices. The point of sale was an important cue for sustainable choices. Participants perceived food as healthier, more environmentally friendly, and beneficial for local producers when it is bought from alternative retailers like farmer markets—or even from a supermarket’s counters instead of off the shelves: “*Yes, it’s nicer, sometimes it’s practical, it’s nice, somewhat more natural*” *[Jennifer]*. Food produced by big food companies was perceived as unhealthy and unethical: “*There is always on Facebook you should not buy Nestlé, Danone […] all those gross companies*” *[Jennifer]*. Highly processed food like ready to eat meals, fast food, and other convenience food was perceived as less healthy “*I’d like to discuss this [organic] yogurt, because I have an aversion to fruit yogurt. I prefer buying plain yogurt, take some strawberries and put fresh ones in it*” *[Selda]*. A healthy diet is also associated with natural ingredients, homemade dishes, and the absence of specific ingredients. Surprisingly, these ingredients varied amongst participants. Selda remained vague, mentioning only “*all those additives*”; Jennifer was concerned about “industrial” sugar and Margot utilized unhealthy fats as a cue for (un)healthy food. Jennifer was more concerned about the health effects of dairy or wheat products. Furthermore, all participants perceived a reduced meat consumption as being sustainable—mainly because of a lack of acceptance of current livestock farming but also because of health and environmental aspects: “*Because eating meat is not that great in general*” *[Laura]*. The degree of packaging—especially plastic—served as a cue to derive the environmental friendliness of a product: “*Because I saw a documentary recently, about the oceans and all the plastic things that are in there*” *[Selda]*. Figure 4 shows which product attributes and consumption practices participants associated with sustainability values.

However, the connection between the mentioned attributes and practices and the desired values was rather loose and based on simplifications and mental short cuts like: ‘organic is better’, ‘the nearer the better’, ‘small scale producers are sustainable’, or ‘cheap meat is not good’. It seems that those simplifications enabled participants to evaluate which products they perceive as sustainable, regardless of their actual knowledge about the underlying standards of organic production or their knowledge about the environmental impact of certain food characteristics or distribution channels. Especially the terms ‘local’ and ‘organic’ were used rather as umbrella terms used to classify products without consciously knowing specific characteristics. This led to a vague feeling, or simply to the hope, that something might be better, instead of certainty about why some choices would be better than others with respect to sustainability. Thus, relying on those attributes did not necessarily simplify choice, but could make it even more challenging, especially, when sustainability attributes conflict with each other, for instance, when confronted with a plastic-packed organic cucumber or an organic apple from New Zeeland. In such cases, the fact that not all attributes are consistent with the desired value leads to uncertainties and frustration: “*It is nonsense; I buy organic apples, but the organic stuff has been flown in from I don’t know which country*” *[Laura]*. Such encounters left participants unable to decide which attribute (e.g., organic production methods, short transport distances for regional products, less packaging waste) may contribute more to the desired value (environmental friendliness):“*I was really angry last time and put it [organic cucumber] down, because I thought ‘you are not doing this, I am not buying a cucumber that is completely packed in plastic*” *[Selda]*. However, it was not only attributes that can compete: sustainable consumption practices also conflicted with sustainable product attributes. Jennifer, who likes shopping at from an elderly farmer at the farmers market in a nearby city, for example, complained that: “*He cultivates according to moonlight, but he puts your salad into a plastic bag, that bugs me*” *[Jennifer]*.

To successfully implement a diet they perceive as sustainable, participants stated that they actively try to avoid/limit/substitute certain products or ingredients, for instance, by eating less animal based products, avoiding sugary products and packed convenience food. However, this strategy often conflicts with old habits and with the preferences of other household members: “*Oat milk and such stuff, we tried different things but nothing worked*” *[Laura]*.

Additionally, the relevance of organic in participants’ choice seems to be restricted to certain product categories. Participants stated that they buy organic products regularly in the case of lower-priced products like eggs (Selda, Laura, Margot, Julia), milk (Julia, Selda, Margot) and yogurt (Laura, Margot). In the case of butter, meat, vegetables, and fruits, they only occasionally chose organic: “*Concerning meat not strictly” [Holger]*. For cheese and other processed foods like pasta or chocolate, organic production methods were less relevant: *“For cheese, I don’t pay attention [to organic]*” *[Laura]*.

Another (unconscious) strategy was choosing products which are “satisfying”—rather than optimal in terms of sustainability—by choosing comparatively cheaper products, still matching sustainability values. During one of our shopping trips, Selda, for example, looked at organic eggs and said: “*Those are organic eggs, 6 pieces. I don’t know how much they are. 2,49€! That’s too expensive!*” *[Selda]*. This is particularly interesting because in our interviews, she indicated that she exclusively buys organic eggs. However, later, she explained that she buys the cheaper ones from the discounter and not the expensive ones with a Demeter label. This was a common phenomenon while discussing several organic labeling schemes. The image of being ‘close to nature’, and therefore better in terms of sustainability, was more intensive for products with labels from farmer associations, whilst products labeled with the European logo were associated with mass production and bureaucracy: “*That one [EU organic] I associate with the EU*” *[Selda]*. However, all participants admitted that products bearing organic labels from farmer associations like Demeter do play a minor role during everyday food purchase because they are too expensive to fill the whole basket with. Whilst shopping at the organic farmer’s shop, Margot, for example, said she “*doesn’t see why*” she should buy the most expensive organic brand. In general, our findings show that food quality labels, which—in theory—should build trust and signal sustainability, were not very meaningful for our participants. Despite trusting the labels and the respective institutions and associations, they were rather irrelevant during food shopping: “*I just look at organic or non-organic*” [*Jennifer]*.

At the point of sale, participants choices were rather influenced by the choice architecture, meaning the way choices were presented to participants: Julia and Laura mentioned that shopping at organic supermarkets facilitates choice: Here, they perceived all products sold to be sustainable, and they could focus on other cues, e.g., the price: “*If I buy at the organic [super]market, I am the holy mother anyway and I can take every product. Then I do not need to focus on local or organic*” *[Julia]*. Laura, Nina, and Julia used product prices as a reference point for product quality; while they avoided choosing the cheapest product, they also refrained from picking the most expensive ones. It seems that such strategies enabled participants to make satisfying compromises between acting according to their sustainability ideas and values, easing the complexity of choice, and ideally restricting their financial expenses. Instead of official labeling schemes, participants relied on the retailers’ organic labeling schemes (Julia, Selda. Laura, Jennifer, and Nina), not because they were perceived as more trustworthy but because they had higher visibility. Furthermore, they relied on the variety of the assortment to assess when fruits and vegetables are in season (Laura), on product prices (all participants), and the arrangement of products (Laura and Julia). For example, Julia went for products the retailer highlighted by arranging them on separated stands indicated as “local corner” and was able to restrict their choice to the products in that stand.

### 4.5. Summary: Reasons for the Attitude-Behavior Gap

Overall, the results revealed that participants are flexible when it comes to sustainable choice and that the determinacy of positive attitudes is highly dependent on external and situational factors. Based on our findings, we suggest that there are four main reasons why our participants acted contrary to their positive attitudes and behavioral intentions:

- Sustainability values conflict with personal, intra-family, external, or situational influences, e.g., household realities, a lack of time, an unsatisfying assortment, habits and traditions;
- Values not concerned with sustainability outdo sustainability values;
- Sustainability attributes/practices conflicting with each other leads to uncertainties and frustration;
- Contradicting messages and a lack of feasible information aggravate the ability to evaluate and identify sustainable products.

### 4.6. Dealing with Inconsitencies

Participants occasionally neutralized and justified their inconsistencies by blaming retailers and producers, e.g., for selling almost all products within plastic packaging (all) or for failing to offer an affordable more sustainable alternative (Selda). Laura complains that even the (presumably sustainable) producers sell their products in a plastic bag, and Selda declared several unsustainable choices as “*exceptions*”. Nina justified buying exotic fruits with “*We already don’t own a car*”. However, participants did not always consciously recognize their unsustainable behavior or, at least, lacked an explanation for it. Jennifer, for example, did buy the organic cucumber packed in plastic during one of our shopping tours, despite complaining about the plastic when she saw it. Margot bought a mango from Brazil to prepare a fruit salad for our second visit, although she mentioned that she never buys exotic fruits. During most encounters, however, participants were aware of and admitted to inconsistencies in their shopping behavior, and there was no need for them to justify their actions. “*I realized it is ok to do this [shop at the organic supermarket] in my restricted possibilities […] I don’t try to drive to the organic supermarket for every shopping trip. I can only make it on Saturdays and otherwise, I go to the supermarket because its faster*” *[Laura]*. “*Well, I am not an organic god, not by any means*” [Selda]. I think I told you I buy local fruit, but that’s bull****. At the moment I buy a lot of kakis because there are a lot [in the supermarket] right now. But those are not German kakis of course. That’s silly” *[Julia]*.

**Figure 5:**
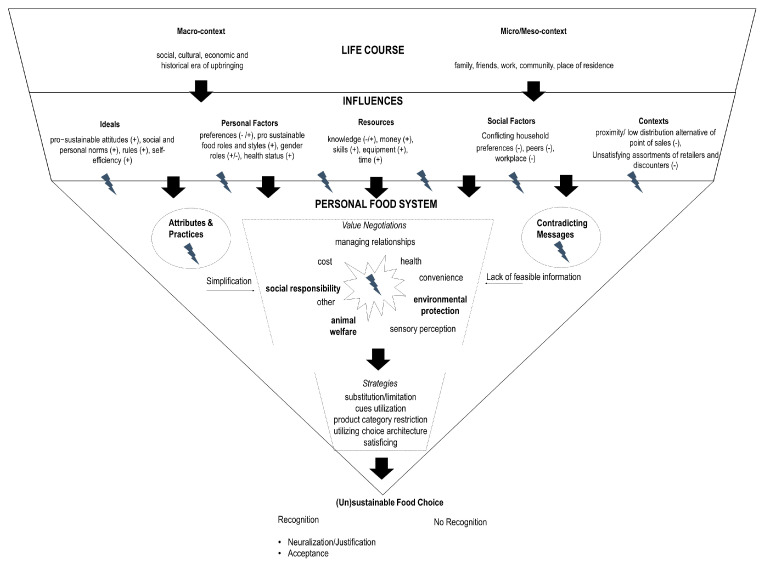
The food choice process model for consumers involved with sustainable food choice based on the model from Furst et al. (1996) [51] and Connors et al. (2001) [60].

Figure 5 shows the Food Choice Process Model adapted for sustainable food choice. It specifies those components of the food choice process that influenced the translation from attitudes to behavior within our sample and indicates where conflicts were most likely to appear.

## 5. Discussion

Participants’ reports were consistent with the existing literature regarding sustainable consumption arguing that positive attitudes [8,9,14,68,69,70] social and personal norms [40,71,72,73,74,75], altruistic values [69,76], and perceived effectiveness of individual behavior [25,40,76]do play an important role for sustainable purchase intentions and behavior. The addition food values emerging from the study animal welfare [77,78] environmental protection [8,79], and social responsibility [80] have gained attention in previous research concerning sustainable consumption, and together with the value health, they cover different sustainability dimensions. However, considering the method used, it does not come as a surprise that there were inconsistencies and that a positive attitude was rather a necessary but by far not a sufficient condition for sustainable food choice. Broadening the research focus and moving beyond the role of intrapersonal factors allowed this research to acknowledge that food choices are influenced by a multitude of factors that mutually, reinforce, interact, and compete with one another [51] and that shifting consumption patterns toward being more sustainable seems to be more complex than changing attitudes and intentions.

The models’ life course perspective enabled this research to understand that pro-sustainable attitudes and practices are not static but emerge and develop within the temporal, social, cultural, and historical contexts. In line with previous research concerning the attitude-behavior gap, our findings emphasize that sustainable habits build over a lifespan [81,82]. However, food habits are a product of ecological forces acting within the context of historical conditioning and cultural belief systems [83], and as part of this dynamic process, they undergo changes. While, participants’ food practices, as most food habits, were formed in early childhood [53,54,84], sensitive periods of life had the potential to change food trajectories. As other research pointed out earlier, pregnancy [85] and the presence of (young) children [86,87] as well as illnesses [88] represented such turning points promoting participants sustainable food choice behavior. However, smaller events as entering the job market and relocations could also induce changes.

While the literature suggests that forming food identities that incorporate pro-sustainable ideals, e.g., being a vegan can be reliable means of overcoming the green gap [30], participants indicated that maintaining integrity within such roles throughout daily life is challenging. Additionally, traditional gender concepts and practices of domestic-work division are still very resilient in Germany, even in female-breadwinner families [89]. Not only were our female participants more involved with sustainable consumption [69,90] and primarily responsible for grocery shopping [91], but they willfully took almost full responsibility for care work. Within this role, they find themselves within a dilemma: while the presence of (young) children fortifies the role as “caretaker” and thus reinforces pro-sustainable ideals and self-efficiency and career opportunities increase the availability of financial resources, which facilitates implementing a sustainable diet, balancing caretaking and job responsibilities is perceived as stressful and leads to a shortage of time.

Although the relevance of time is seldomly mentioned in rationalist research concerning the attitude-behavior gap (Young et al. (2010, p. 30) [92]) already pointed out that “‘being green’ needs time and space in peoples’ lives which is not available in increasingly busy lifestyles”, and time barriers [93], e.g., limited discretionary time and longer purchase time [94], can promote the attitude-behavior gap significantly.

It could be this time shortage together with the food context, specifically the point of sale, within the immediate neighborhood that contributes to a perceived “lack of availability”, one of the well-documented barriers for sustainable food consumption [14,95]. According to the literature, it is important that grocery shopping fits in with daily routines and practices [96,97,98,99], otherwise consumers may sacrifice sustainability values to the convenience of a one-stop-shop [32]. While participants associated sustainable consumption with certain grocery shopping practices, e.g., shopping at organic supermarkets, farmers markets), primarily because they are not completely satisfied with supermarkets and discounters’ assortment with respect to sustainability, performing those practices requires additional time and in daily life visiting a nearby or well-known supermarket seems to be more convenient and safer concerning the assortment.

Habits do play another important role in driving consumers towards a particular purchase [81,100]; however, breaking old habits and forming new ethical shopping habits require an effort beyond ethical product selection [101]. Our findings suggest that participants’ personal preferences, especially personal biases, and cravings are deeply internalized and can be rather hard to overcome, despite sustainable ideals and behavioral intentions.

Furthermore, whenever consumers perceive the quality of sustainable alternative inferior to the non-sustainable alternative, this promotes the attitude-behavior gap significantly [23,34,43,44,94]. In our study, this was usually the case for sensory perceptions and taste in particular. Taste acted like a gatekeeper: when food is not tasty, no other value could compensate for this fact, and therefore, the product will not be bought. Considering that food behavior is heavily influenced by social factors, aggravates this aspect. When eating with the family or peers, the needs and preferences of other people have to be negotiated [102] especially with those from other household members [51,64]. Hereby, children’s needs seem to be the most determinant. Not only did the children in our study affect parents’ decisions by communicating their food preferences [103], but according to their parents, they usually simply refuse to eat food that they do not like. Thus, inconsistencies were likely to appear, if household member preferences were not reconciled with the sustainable products, or choosing the sustainable product would threaten the harmony within the household or endanger child nutrition.

Concerning the role of knowledge, it was not necessarily a lack of knowledge that was a barrier for participants sustainable food choice [14,95,104], but rather, it was contradicting (media) messages and a lack of trust in the reliability of information that diminished its usefulness. Instead of enhancing self-confidence and facilitating the translation of attitude and motivations into behavior [40,42,86], this kind of subjective knowledge was not always able to reduce participants’ uncertainties and could even lead to more confusion in choice situations. Carrigan and Attalla (2001) [23] already argued that so-called sophisticated consumers have so much knowledge today on consumer products that it can actually detract from, rather than enhance, choice, a problem which has very likely increased with social media. The majority of the literature suggests that informing consumers can bridge the attitude-behavior gap [38,40] and that trust in official organic labeling schemes plays an important role when translating attitudes into intention and behavior [39]. However, in line with previous qualitative research on the issue [23,92], our findings suggest that in everyday life, consumers have no time for extensive information research.

Surprisingly, providing participants with information about food labels did not inevitably affect choice either. A reason for this might be that behavioral change does not depend on exposure to information alone, but individuals need to make connections between their knowledge and behavior and the broader environment [105]. While the literature argues that consumers may not recognize the consequences of their food choices and are not confident about the efficiency of individual actions [24,25], this did not apply to our participants. Though, they were confident about the efficiency of their behavior and trusted official labeling schemes, during our shopping trips, official food labels were rather irrelevant. According to Osman and Nelson, (2019) [106] it might be that public information campaigns (e.g., Food Quality Schemes) are too localized to provide a salient connection between individual actions and meaningful changes with respect to sustainability in the short and long term. Thus, they are unable to reduce uncertainty and increase personal agency around the impact of behavioral changes, which in turn might limit the causal impact they might have.

While our observations are consistent with the existing literature on sustainable consumption arguing that higher costs are a crucial purchase barrier [40,107,108], sufficient financial resources are a precondition for actual choice [109], and food prices seem to be more determinant in earlier stages of life [64], the impact of comparatively higher prices for sustainable foods seems to be more complex. Even in later life stages and within a comfortable economic situation, participants consider price in the context of disposable income [32] and thus need to balance grocery expenses for weeks or longer. When discussing single products, food prices did play a minor role; however, they did during grocery shopping when confronted with cheaper alternatives. One reason for this might be that it is not the price per se that prevents consumers from purchasing green products, but that price premiums at the point of sale reinforce their perception of green products as too expensive [24,64], and thus, price framing might moderate the relationship between consumers’ perceptions and their buying intention [110]. This might also be the reason why our participants perceive organic and local products bearing retailers’ and discounters’ brands as a satisficing alternative. Simon (1955) [111] already suggested that in choice situations, people rather have the goal of “satisficing” than maximizing. To satisfice, people need only to be able to place goods on some scale in terms of the degree of satisfaction they will provide and to have a threshold of acceptability [112]. It could be that those comparably cheaper products, despite being perceived as less sustainable than products labeled by farmer associations and products from farmer markets, still pass participants threshold in terms of sustainability and, hence, prevent unnecessary expenses.

Previous research already mentioned that food choice is not always rational and deliberated, and consumers used simplifications and decision strategies [48,109,113,114,115,116]. While our participants actively attempted to avoid or substitute certain products or practices, most decisions were rather unconscious and based on simplifications. According to the literature, consumers rely on different cues to facilitate product evaluation [117,118]. However, for our participants, neither sustainability itself, nor the values associated with sustainability were clearly bound. They covered a range of desirable attributes and practices of food consumption which were only loosely linked to specific values. Additionally, attributes and practices associated with sustainability often contradicted each other which led to conflicts and uncertainties. In such cases, as reported earlier by Meah and Watson (2013) [98], confusing messages and a perceived lack of faith in the reliability of information opened up room for the negotiation of ambivalences.

Research concerning the attitude-behavior gap pointed out earlier that consumers are responsive to in-store factors such as displays, signs, and assortments [36,119,120] and that influences at the point of sale e.g., price, quality, and availability [24,33,64] affect sustainable buying behavior. Instead of using official labeling schemes, participants rather relied on the retailers labeling scheme and product prices at a point of sale as an indicator for sustainable products. Surprisingly, modifying the choice architecture by visiting farmers markets and other retailers perceived as sustainable or relying on a certain arrangement of products was a promising self-nudging strategy [121,122] which facilitated participants choice by reducing their cognitive effort and thus was a reliable mean for overcoming the gap.

Using ethnographic research methods, it did not come as a surprise that there were inconsistencies between participants’ statements and their behavior, as concise reflections do bear the risk of overestimating ethical considerations while actual choice is affected by more prosaic factors [123]. Previous research on the attitude-behavior gap for sustainable consumption showed that post-purchase dissonance in the form of guilt at not opting for the ethical alternative is an important aspect of ethical consumption [124] and that consumers apply techniques of neutralization when they do not consider sustainability issues in purchase situations [125,126]. However, in our study, we found that rarely to be the case. Although participants occasionally blamed retailers and producers for packing their products in plastic, and statements as “we already don’t own a car” point towards the phenomenon that sustainable actions can induce rebounds and negative spillover effects on other domains of environmental behavior [127,128], in general, participants talked openly about their inconsistencies and accepted them, without showing much dissonance or remorse. Similar results have been found by Szmigin, Carrigan, and Mceachern (2009) [31] and Moraes, Carrigan, and Szmigin (2012) [30] who reframed the attitude-behavior gap as “coherent inconsistencies”, arguing that inconsistencies between cognitions are not necessarily enough to arouse dissonance. In their review, ElHaffar, Durif, and Dubé (2020) [29] already stated that the dominance of the rational paradigm could be the very cause for the attitude-behavior gap, as it postulates an alignment between attitudes, intentions, and behaviors and presumes that the consumer consciously sees a problem with their behavior and tries to solve it.

Similar to the results from Newholm (2005) [129], however, who found that not all participants within their study sought integrity and consistency and some were happy with, and embraced, the fragmented nature of their behaviors, our participants did not share the researchers’ rational perspective of consistent attitude conform behavior to define themselves as sustainable consumers. Based on those findings, we can describe our participants according to Szmigin, Carrigan, and Mceachern (2009, p. 229) [31] definition of “conscious consumers”: “While the conscious consumer wants to contribute and consume ethically, this does not rule their life and indeed for some, where it is inconvenient, they will not worry about the inconsistencies between their attitude and behavior”.

## 6. Conclusions/Limitations/Implications

Employing ethnographical methods allowed this research a better understanding of the attitude-behavior gap by gaining insights into the phenomenon in its natural setting. Repeated accompaniment and intense contact over months established mutual trust, enabling this research to go beyond the simple inquiry of attitudes and intentions and reflect their development over the personal life course and their determinacy throughout everyday household realities.

The findings show that establishing sustainable consumption practices is more complex than changing individual values and attitudes. Consumers are confronted with a complex and cohesive consumption reality in which the translation of personal pro-sustainable attitudes into sustainable actions is affected by product-related, intrapersonal, external, and situational influences leading to ambivalences, compromises and trade-offs, and inconsistencies. In our study, positive attitudes towards sustainable foods were driven by personal and social norms, altruistic values, and a desire to act morally. However, corresponding behavior was affected by personal preferences, by the availability of resources, and especially by factors at the point of sale and by the composition of the household one is living in. For working mothers, balancing personal and family preferences as well as household and work responsibilities leads to pressure and stress which affects when, where, with whom, and what product to purchase. Additionally, there is no time for studying the vast amount of information, and relying on narratives and contradicting information fragments rather aggravates insecurities. To cope with the demands of this complex task environment, participants employ decision strategies that are not always rational or deliberated but allow them to maintain flexibility.

There is considerable scope in consumer research that remains to investigate the attitude-behavior gap. While this fieldwork represents a case study, it provides useful insights about why consumers involved with sustainability do not always act according to their pro-sustainable attitudes and intentions. To capture the food choice behavior of consumers concerned with sustainability, all the original model’s components proved to be relevant. However, the model needs to be extended by the resource “time” and by the three food values, animal welfare, environmental protection, and social responsibility. Furthermore, the model’s assumptions that value negotiation is a deliberate act and that people negotiate values in food choice according to relatively consistent patterns are questionable. Considering that attributes conflict with each other on many occasions and that there is no solid connection between the food values and respective product attributes and practices, the importance participants attached to different food values is neither consistent nor follows a strict order of priorities. However, the model acknowledges the existence of choice strategies and the fact that consumers behave quite differently in different situations.

To better understand why and when positive attitudes, values, and beliefs are more likely to cause corresponding behavior, it is necessary for researchers to acknowledge that individuals cannot be detached from their household realities and consumption practices. Thus, future research should apply more observational and experimental research methods to investigate the interplay of attitudes, household realities, and influences at the point of sale. In order to gather more information about how sustainable practices can be easily integrated into daily routines, there is a need to develop a richer understanding of those strategies that allowed participants to prevent value conflicts and ease the complexity of sustainable choice. Furthermore, instead of providing more and more information, policymakers and researchers should focus on investigating which information is actually useful to identify sustainable products and reduce uncertainty, how this information can be made easily available, and how people can integrate it in daily consumption practices. Finally, consumer research should refrain from dividing consumers into two groups: those who purchase and those who do not, as with respect to sustainable consumption, the categories of consumers are diverse, having degrees of commitment and showing inconsistencies [29,31,130].

This study has limitations that pertain to qualitative research. The findings are not representative of the German population in a statistical sense. Analyses including other households would differ depending on the region, financial resources, and other socio-demographics. Furthermore, some data have been obtained in a rather unnatural setting (e.g., discussion about the food products brought by the researchers), which is untypical for ethnographic fieldwork. However, especially the initial statement proved to be useful to uncover reasons behind conflicts and inconsistencies when compared with observational data and interview data at later stages of the study (when mutual trust was built). This study took place in one country only. In Germany, discounters and supermarkets are by far the most frequently visited retailers [20]. Hence, future studies might investigate the role of cross-cultural differences within the food context for (sustainable) choice and applied choice strategies.

## Figures and Tables

**Table 1:** Sample of households ^1^.

Household 1	Laura	Adil	2 children
urban	in her 30 s	in his 30 s	male (2 years) in kindergarten
	works in public service	master student	female (4 years) in kindergarten
Recently moved from to North-Rhine Westphalia from Berlin.			
Adil migrated to Germany from an Arabic country ten years ago.			
Household 2	Margot	Holger	2 children
rural	in her 60 s	In his 60 s	grown up, left household
	works for a Christian community	works mostly from home	1 grandchild (1 year)
Holger grew up on a farm.			
Holger had a serious illness in his late 20 s.			
The family regularly orders food from a farmer shop.			
Household 3	Jennifer	Cem	1 child
sub-urban	in her 30 s	in his 30 s	male (2 years) in kindergarten
	works at the bishopric	works in marketing	
Recently moved from the city to a suburban area.			
Cem’s parents migrated from Turkey to Germany.			
Jennifer’s parents migrated from Poland to Germany.			
Household 4	Selda		2 children
sub-urban	in her late 40 s		male (10 years) in school
	school secretary		male (13 years) in school
In a divorce from her husband.			
Recently moved in a new apartment with her children.			
Selda’s parents migrated to Germany from Turkey.			
Household 5	Julia	Frank	1 child
urban	in her 30 s	in his 30 s	male (10 years) in school
	works at a broadcasting company	works at a university	
Julia is vegetarian.			
Frank is not the biological father of the child. Julia and her ex-partner share custody.			
Household 6	Nina	Pablo	3 children
urban	in her 30 s	in his 30s	female (11 years) in school
	social worker	social worker	female (1 year)
The younger female child is Nina and Pablo’s biological child.			
The older female child is Nina’s daughter, she shares custody with her ex-partner.			
Pablo has another son (7 years), and he shares custody with his ex-partner. He visits the household regularly, although he is not a resident.			
Pablo was born and raised in South America.			

^1^ Participants’ names have been pseudonymized.

## Supplementary Materials

The following are available online at https://www.mdpi.com/article/10.3390/foods10061317/s1. Pictures SA: Pictures from ethnographical Fieldwork, SB: Codebook.
