# Supplementary material for: Good Attitudes Are Not Good Enough: An Ethnographical Approach to Investigate Attitude-Behavior Inconsistencies in Sustainable Choice
Source: Foods. 2021 Jun 8;10(6):1317. doi: 10.3390/foods10061317 (PMC8226450; doi:10.3390/foods10061317)
Supplement: Supplementary file 1 [file foods-10-01317-s001.zip › SA_Pictures_from_ethnographic_fieldwork.pptx]

## Slide 1
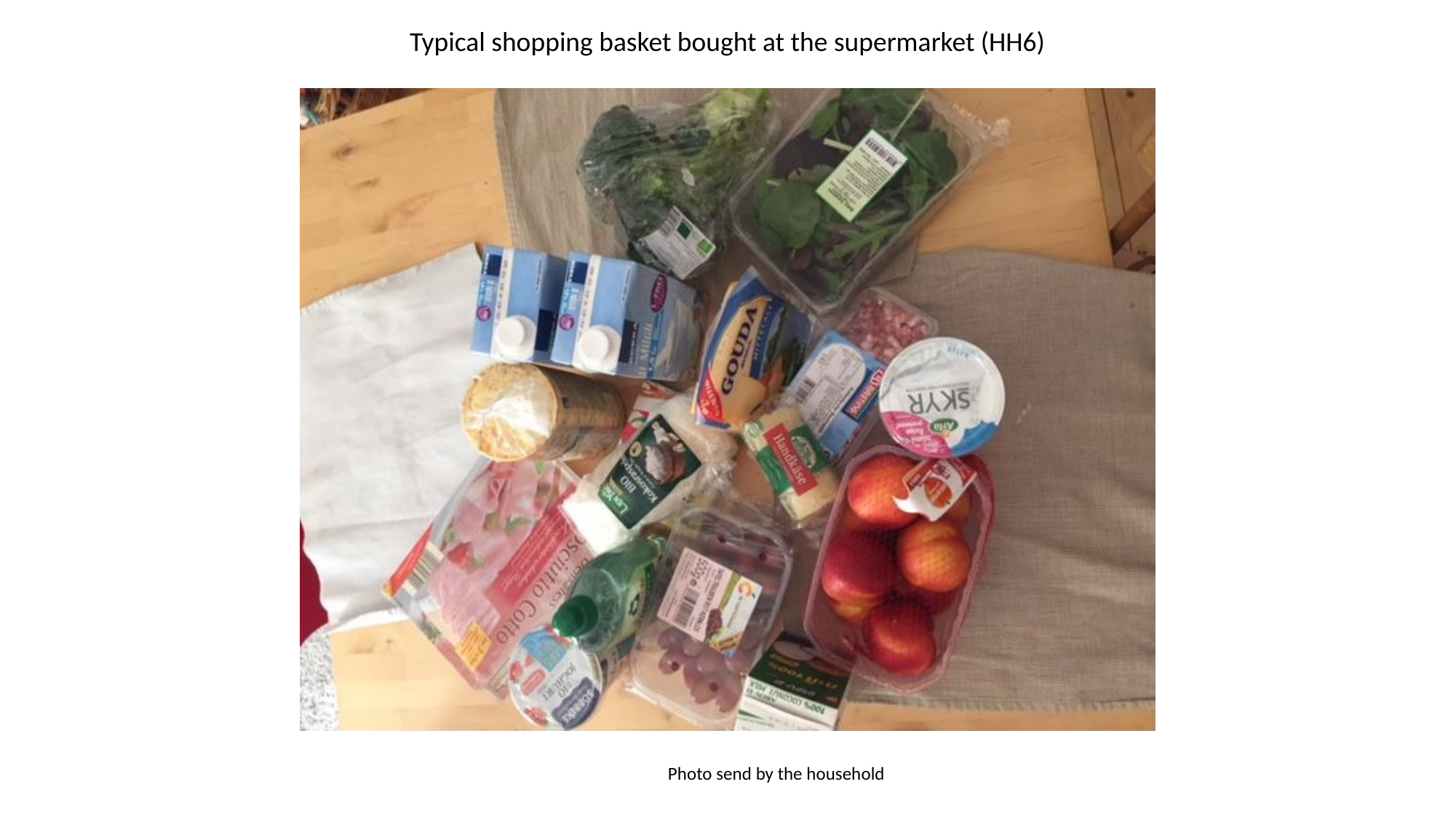

Typical shopping basket bought at the supermarket (HH6)
Photo send by the household

## Slide 2
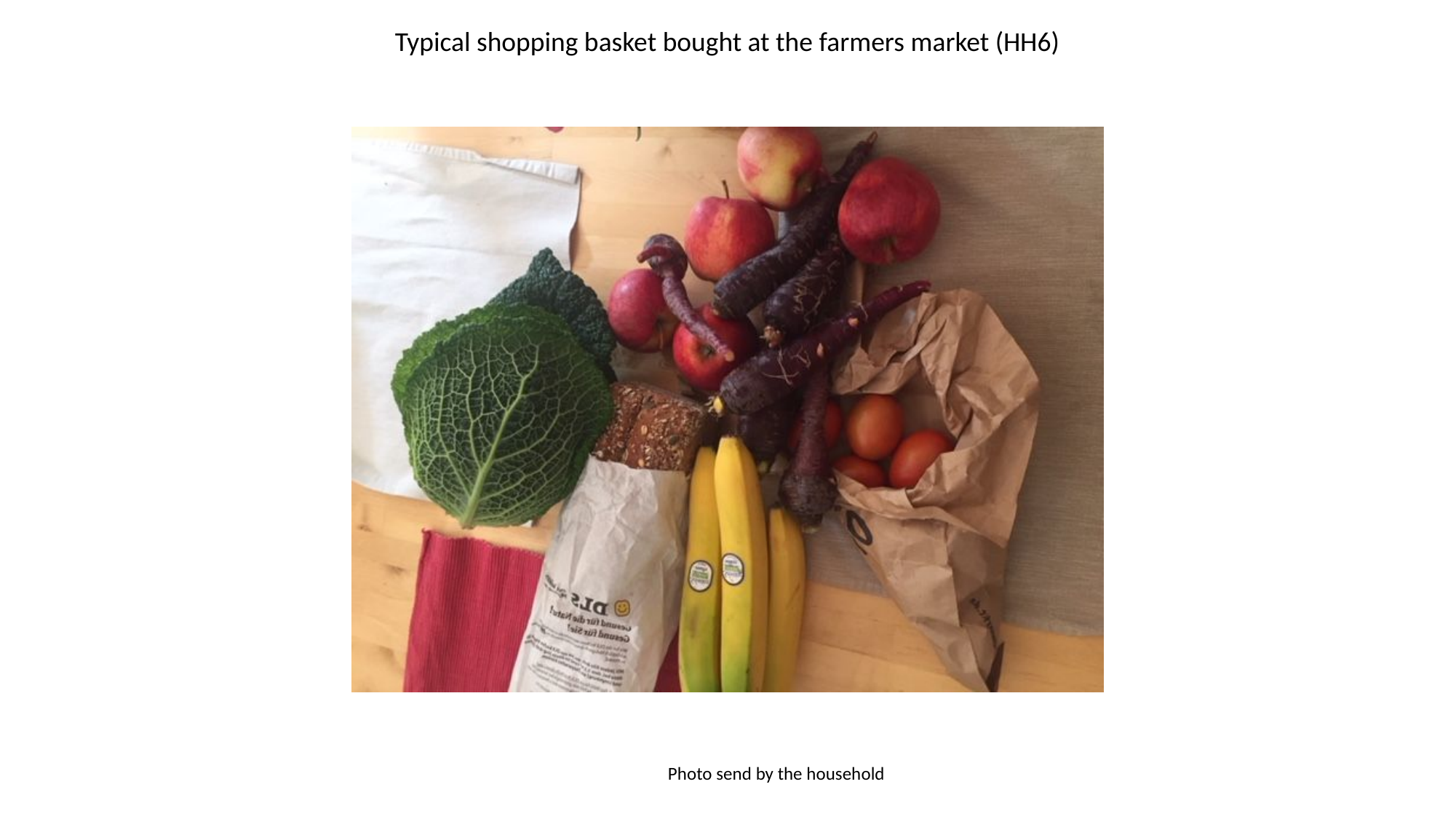

Typical shopping basket bought at the farmers market (HH6)
Photo send by the household

## Slide 3
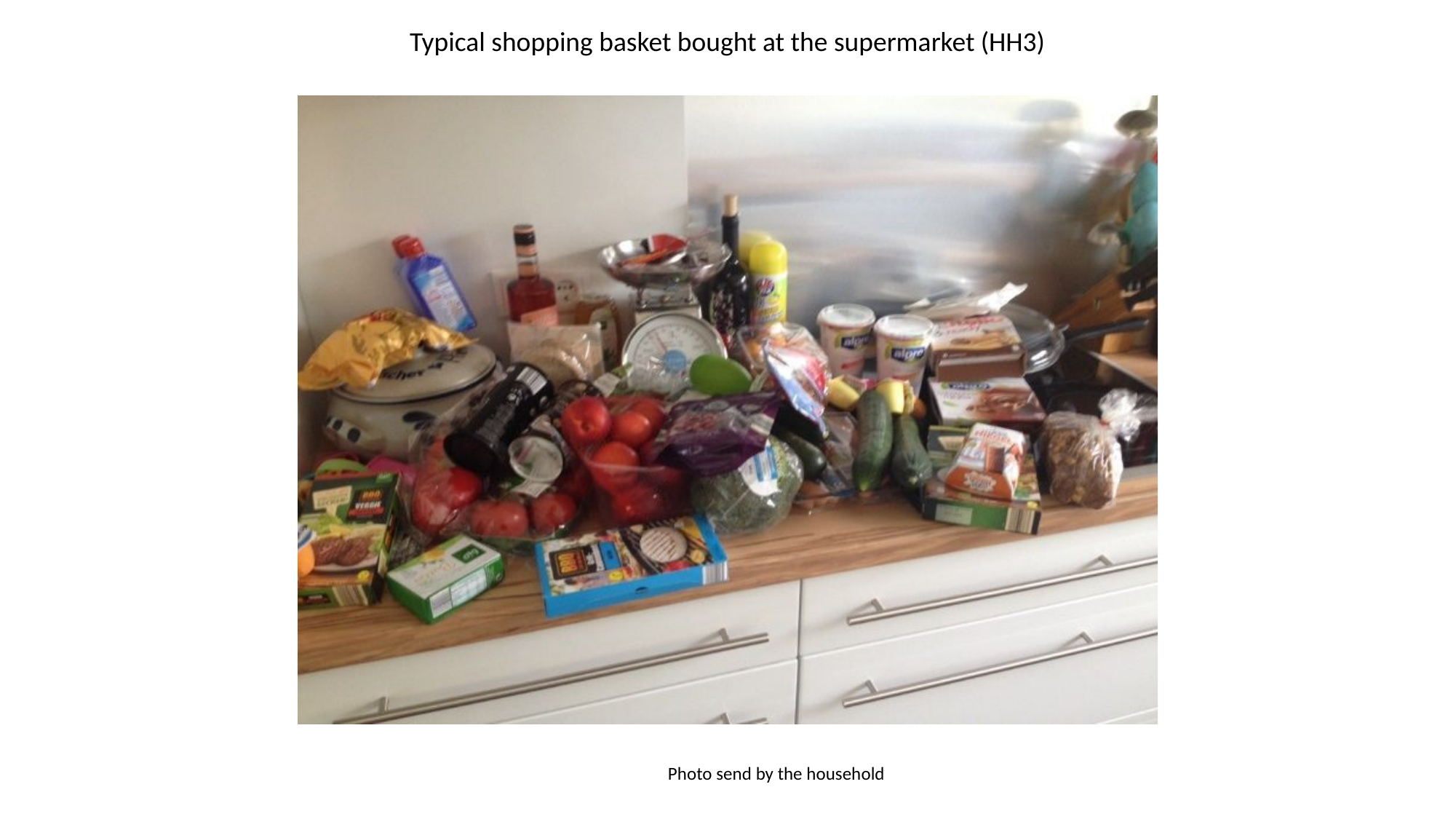

Typical shopping basket bought at the supermarket (HH3)
Photo send by the household

## Slide 4
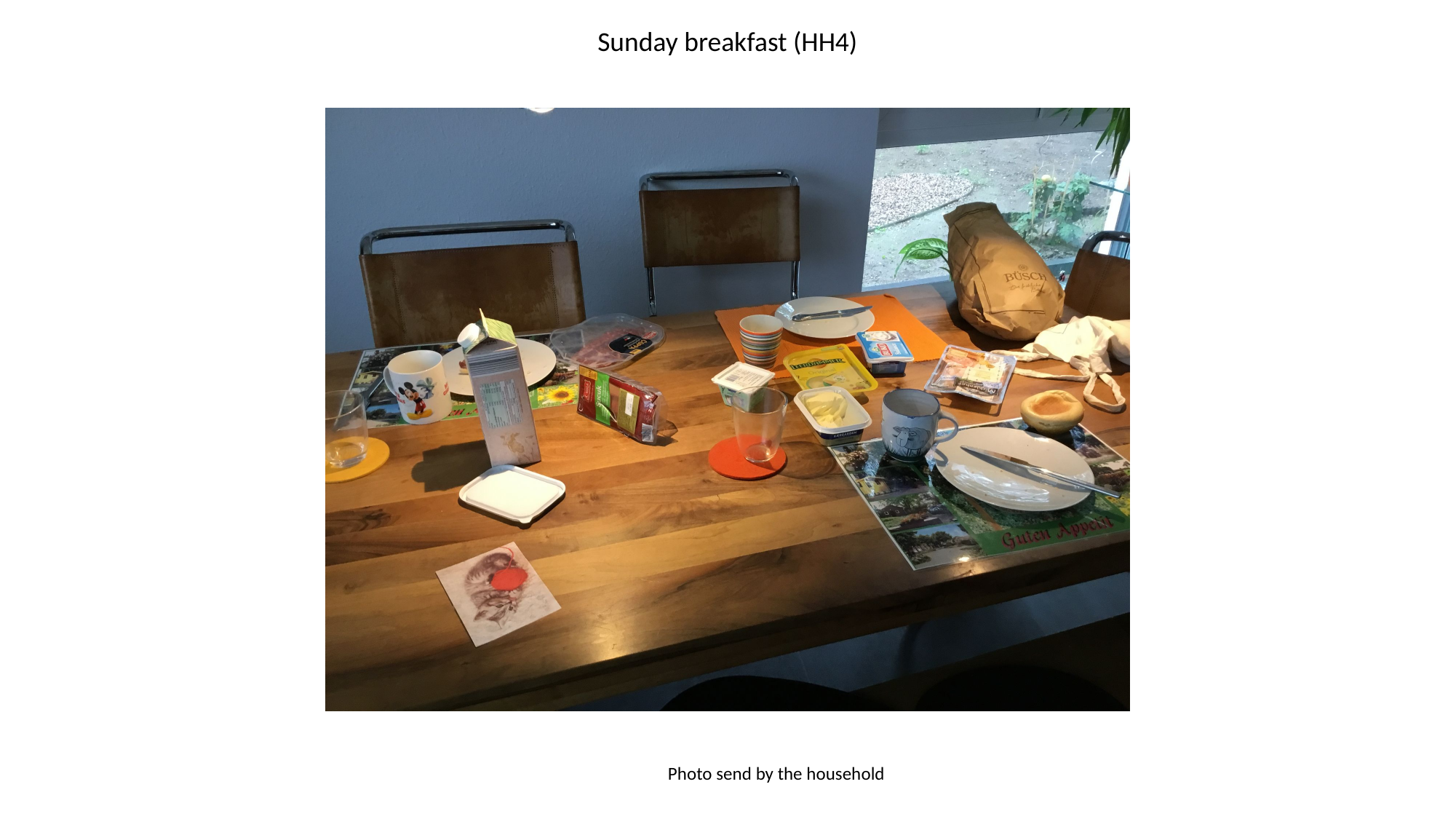

Sunday breakfast (HH4)
Photo send by the household

## Slide 5
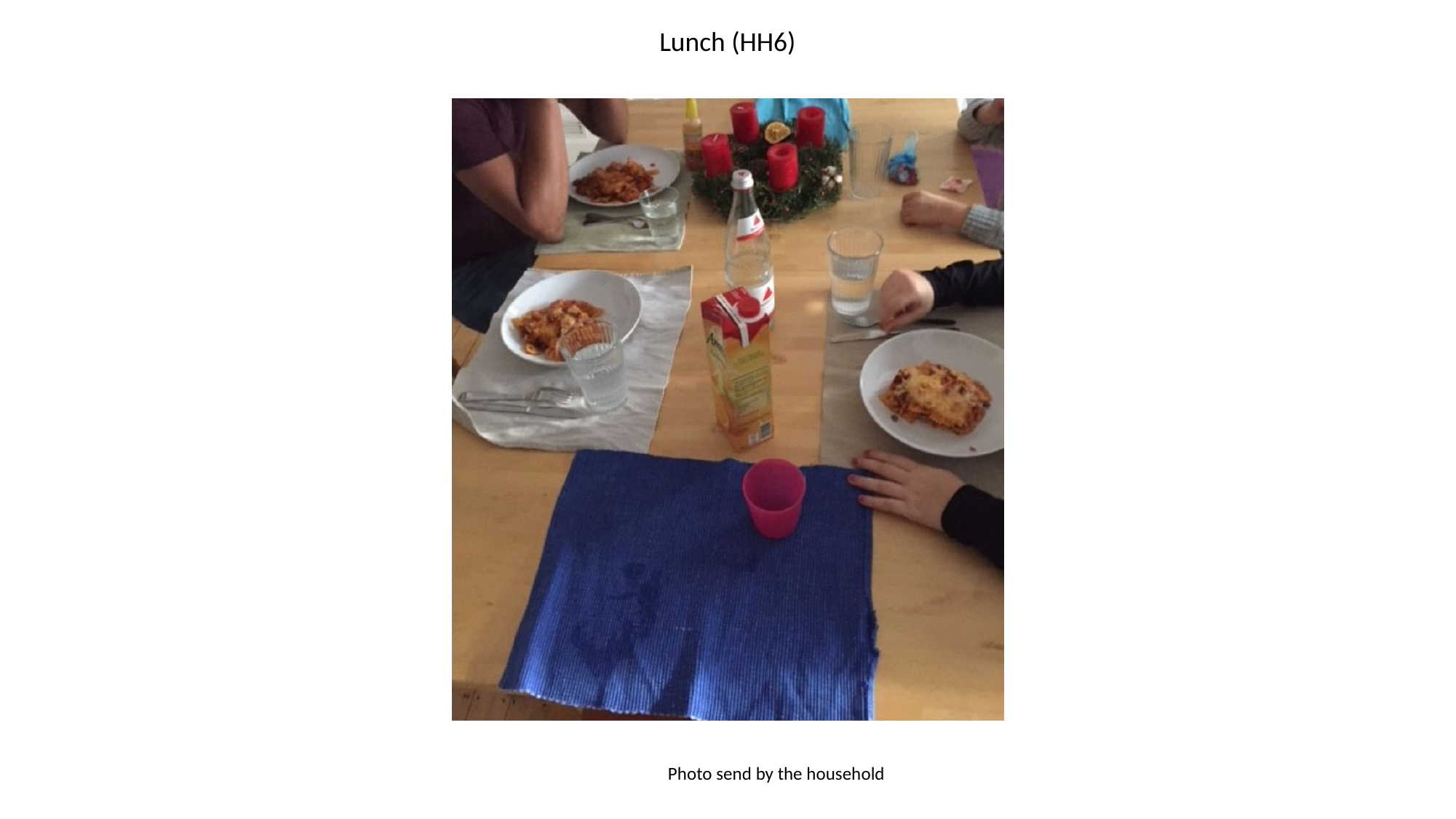

Lunch (HH6)
Photo send by the household

## Slide 6
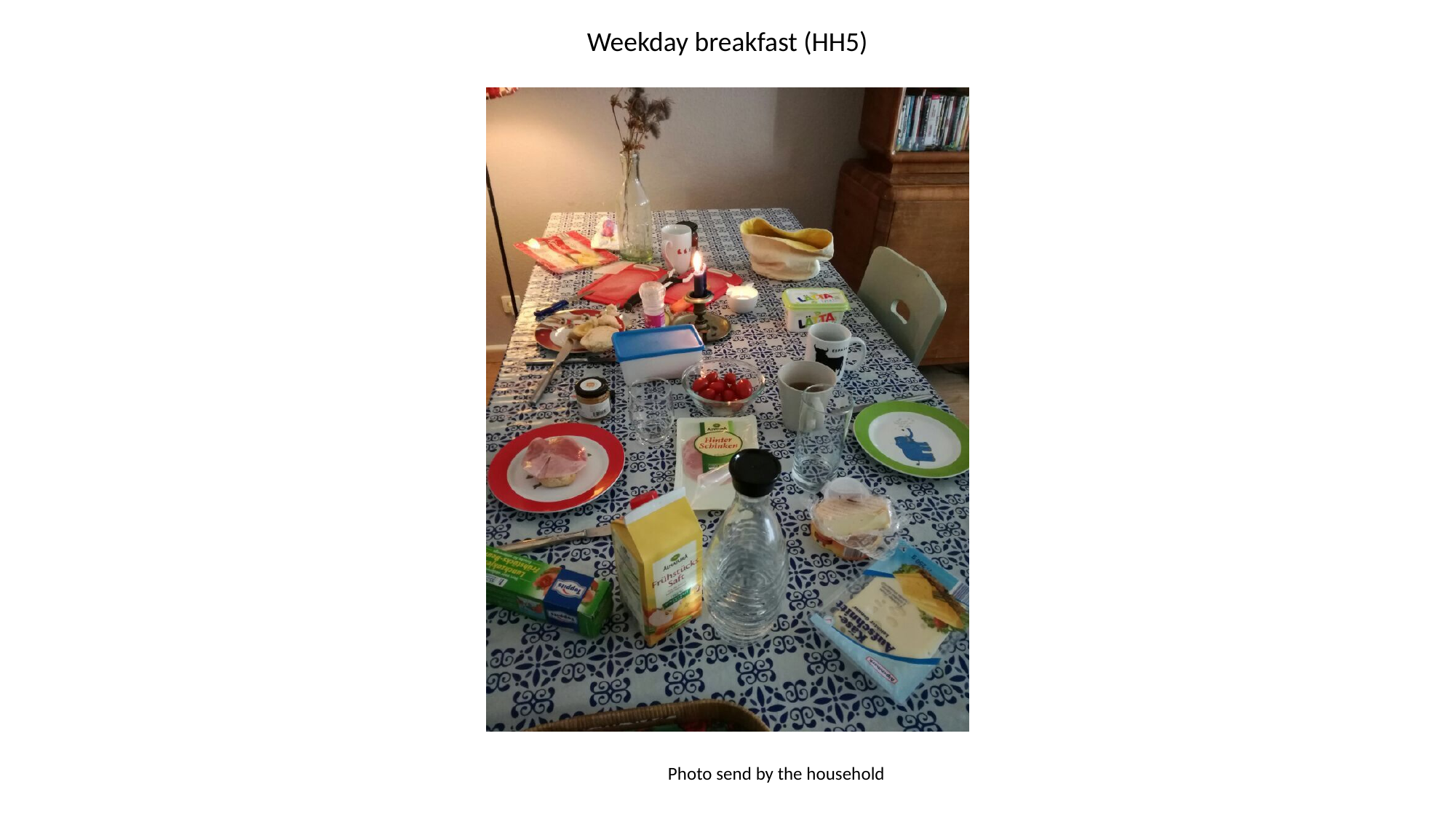

Weekday breakfast (HH5)
Photo send by the household

## Slide 7
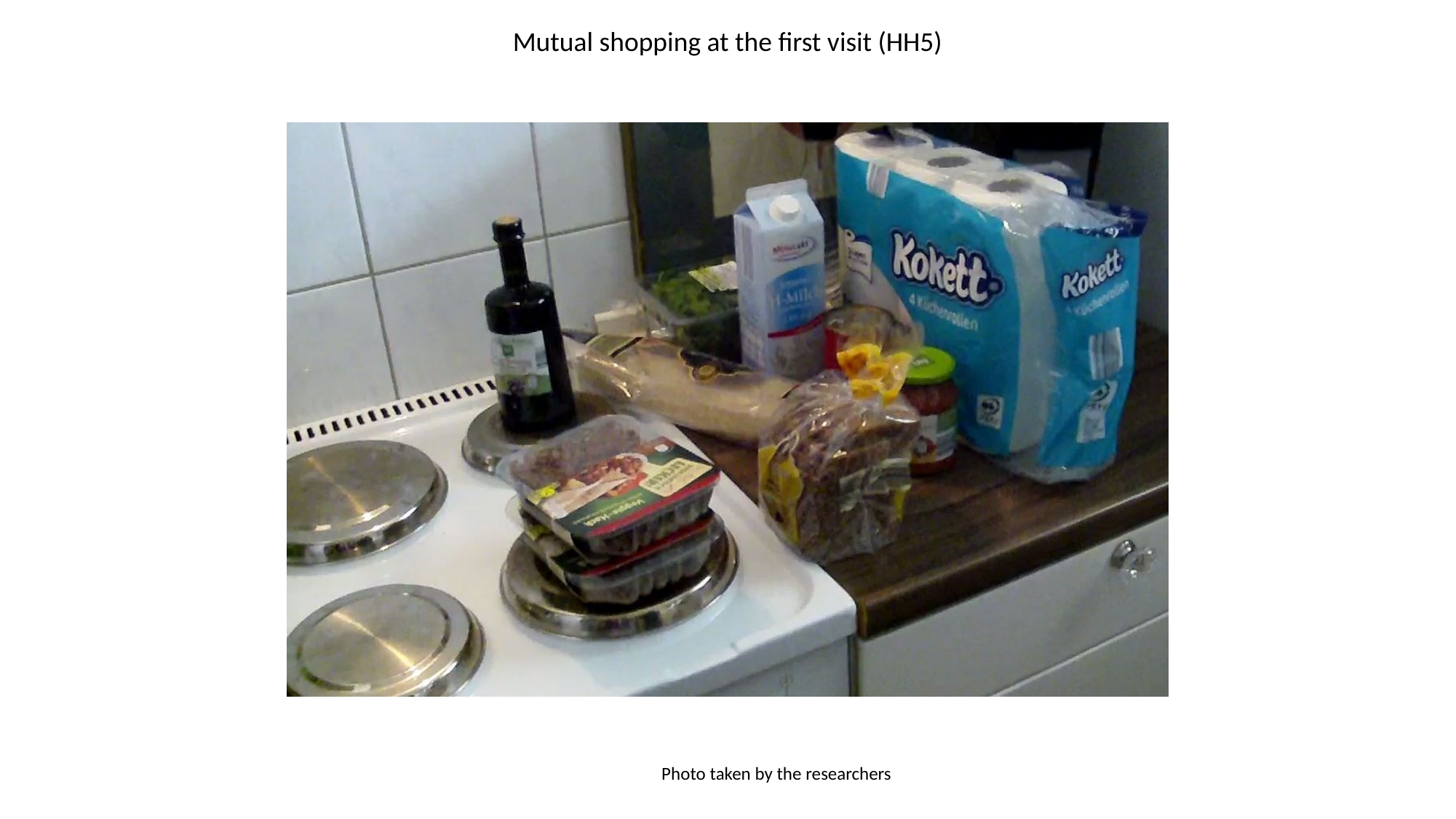

Mutual shopping at the first visit (HH5)
Photo taken by the researchers

## Slide 8
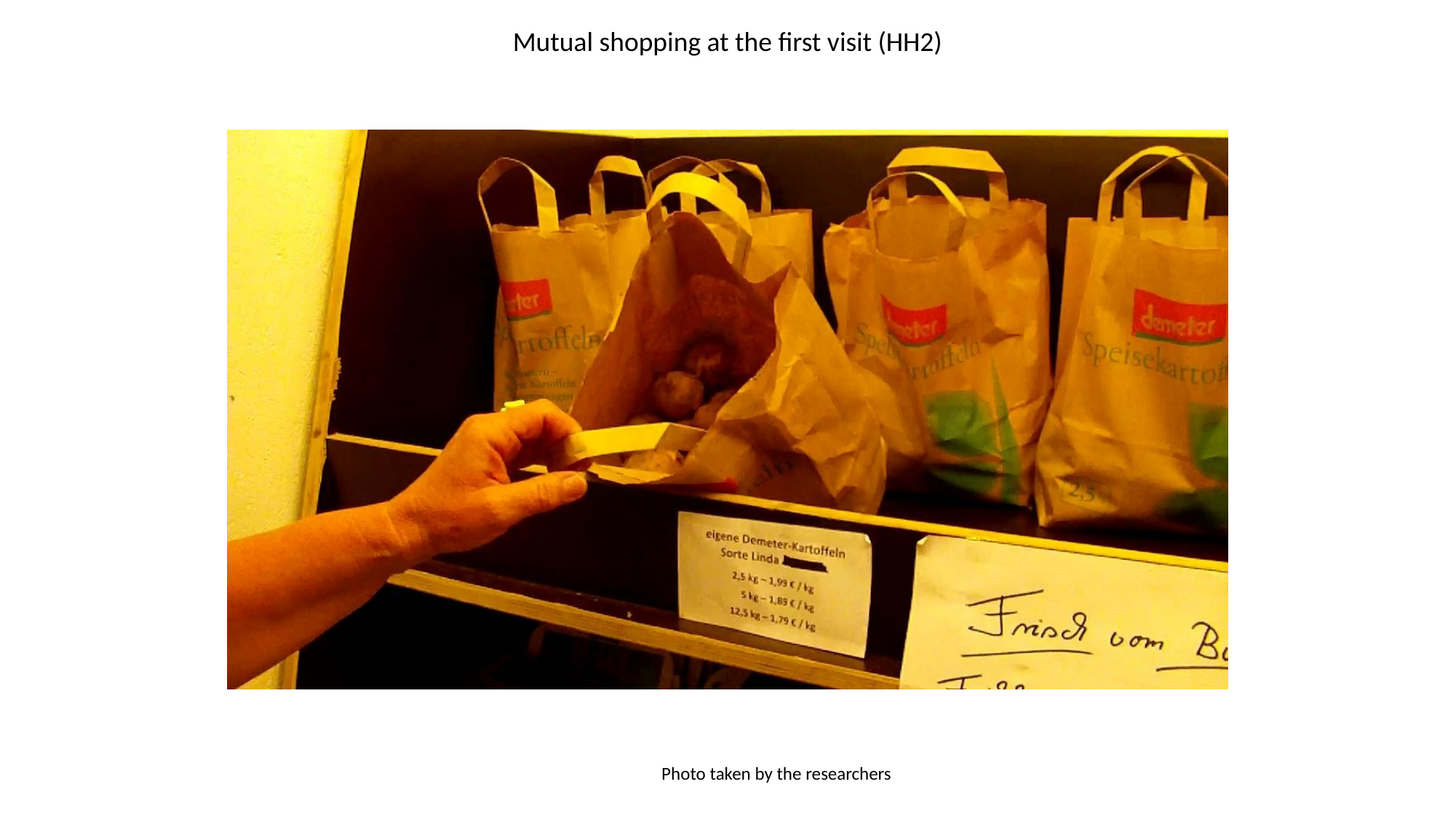

Mutual shopping at the first visit (HH2)
Photo taken by the researchers

## Slide 9
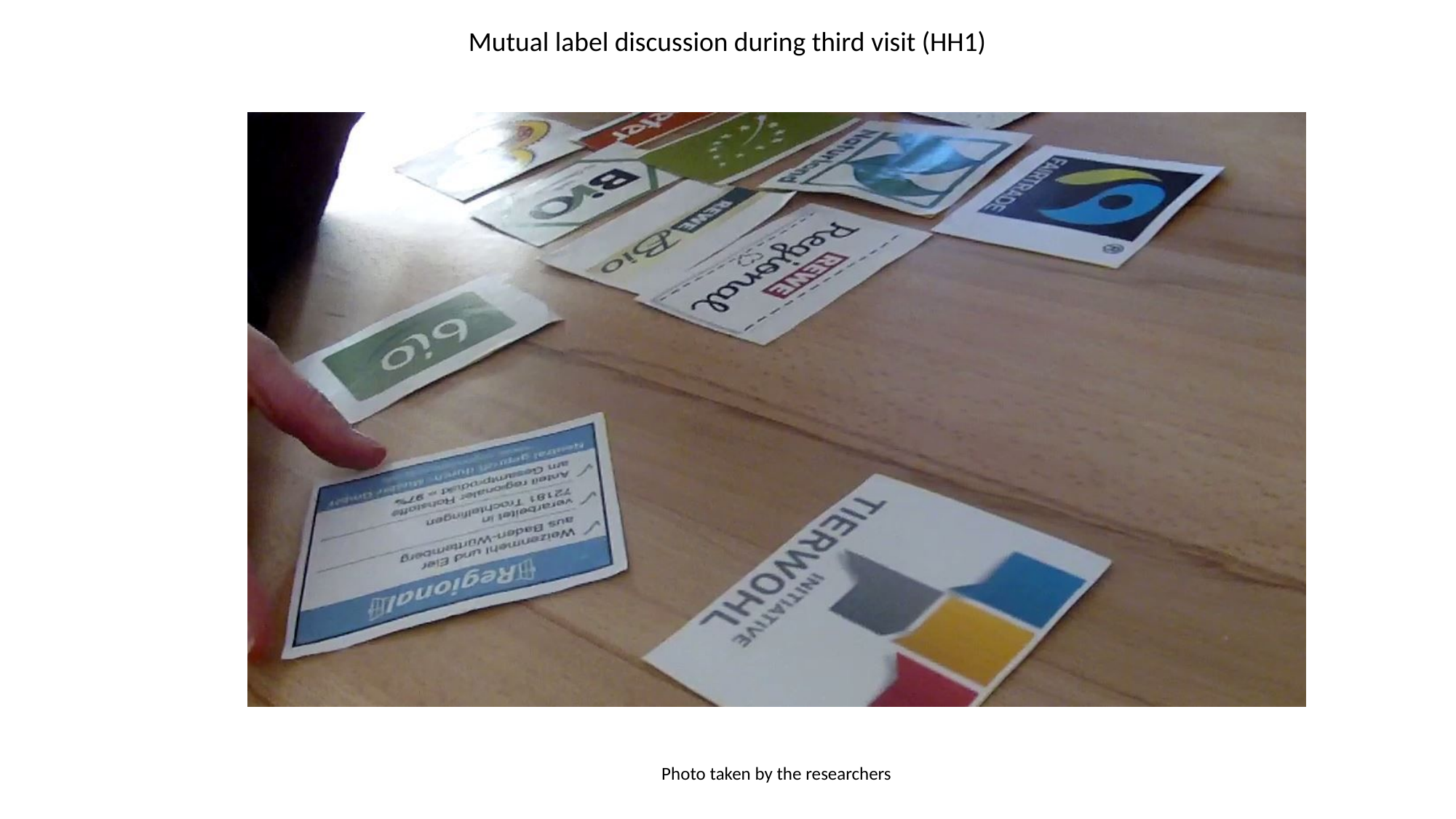

Mutual label discussion during third visit (HH1)
Photo taken by the researchers

## Slide 10
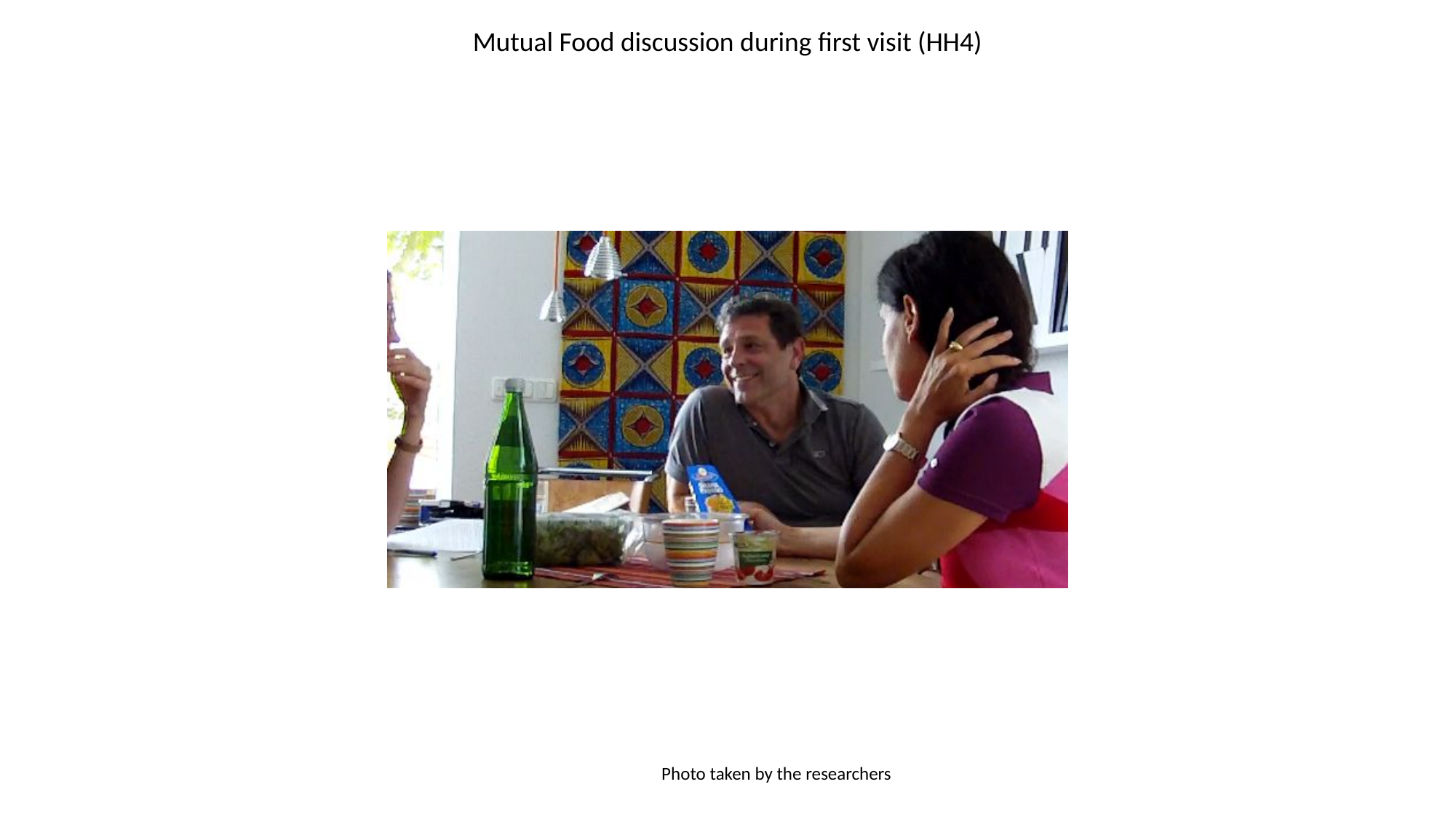

Mutual Food discussion during first visit (HH4)
Photo taken by the researchers

## Slide 11
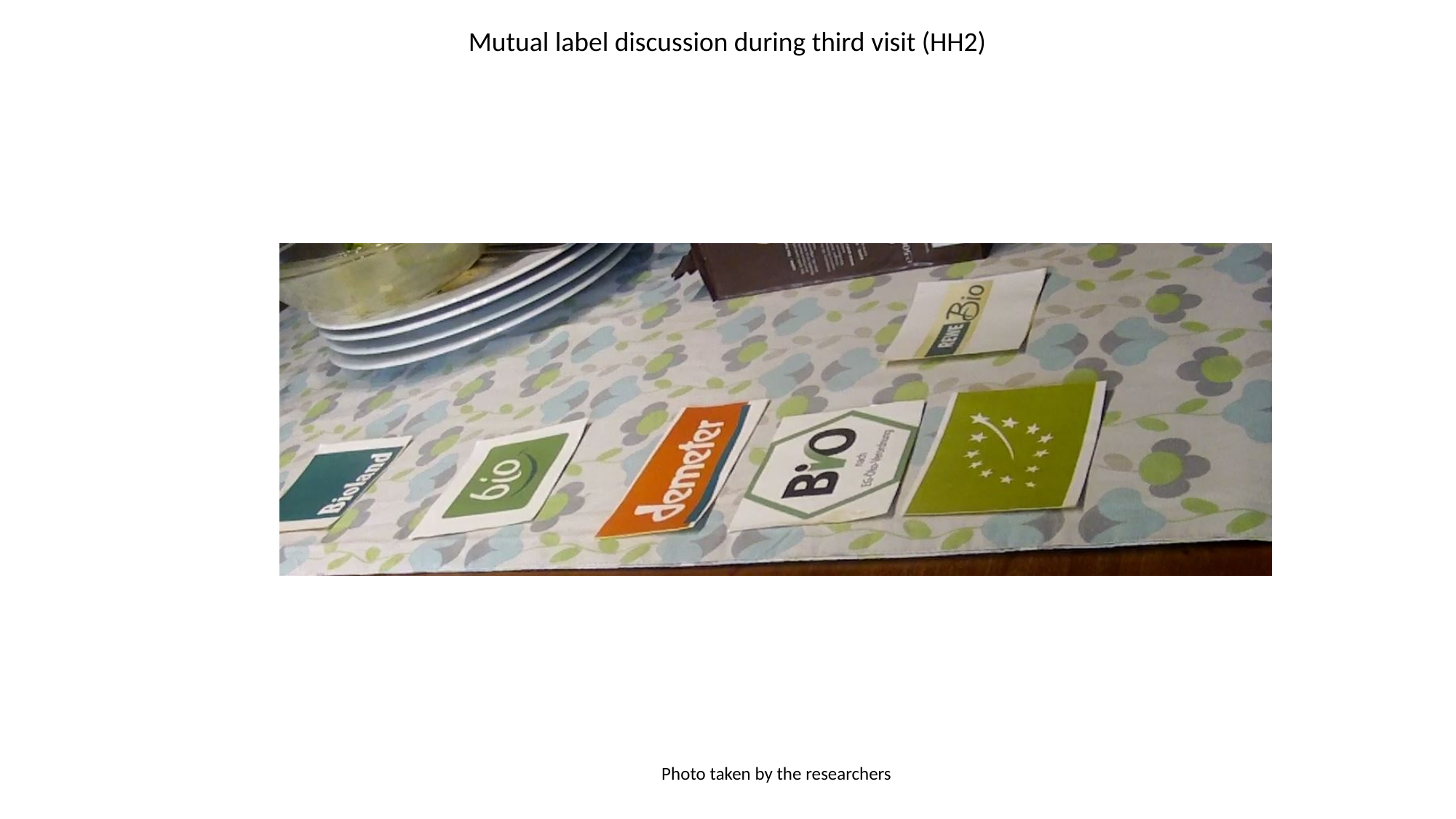

Mutual label discussion during third visit (HH2)
Photo taken by the researchers

## Slide 12
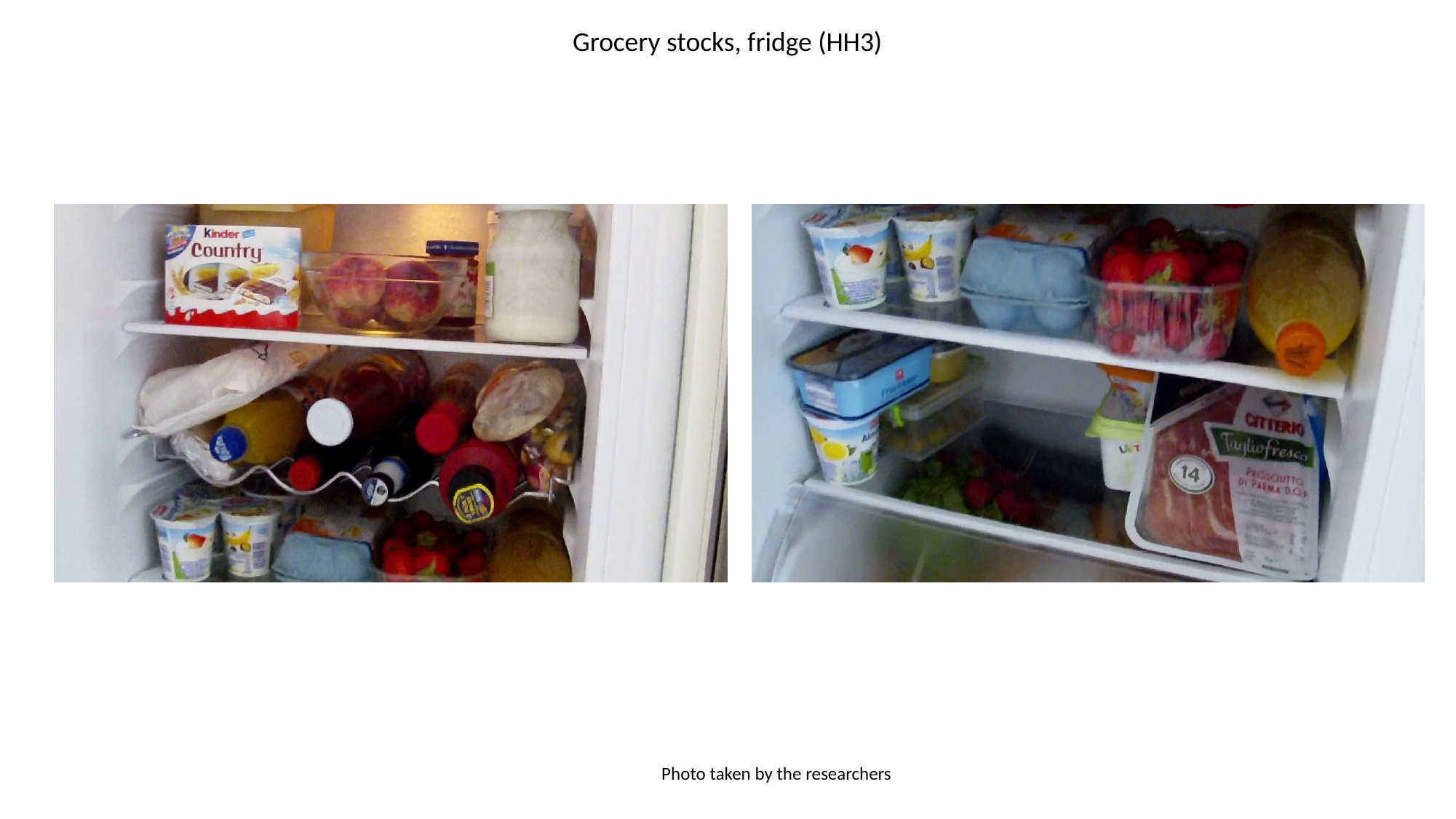

Grocery stocks, fridge (HH3)
Photo taken by the researchers

## Slide 13
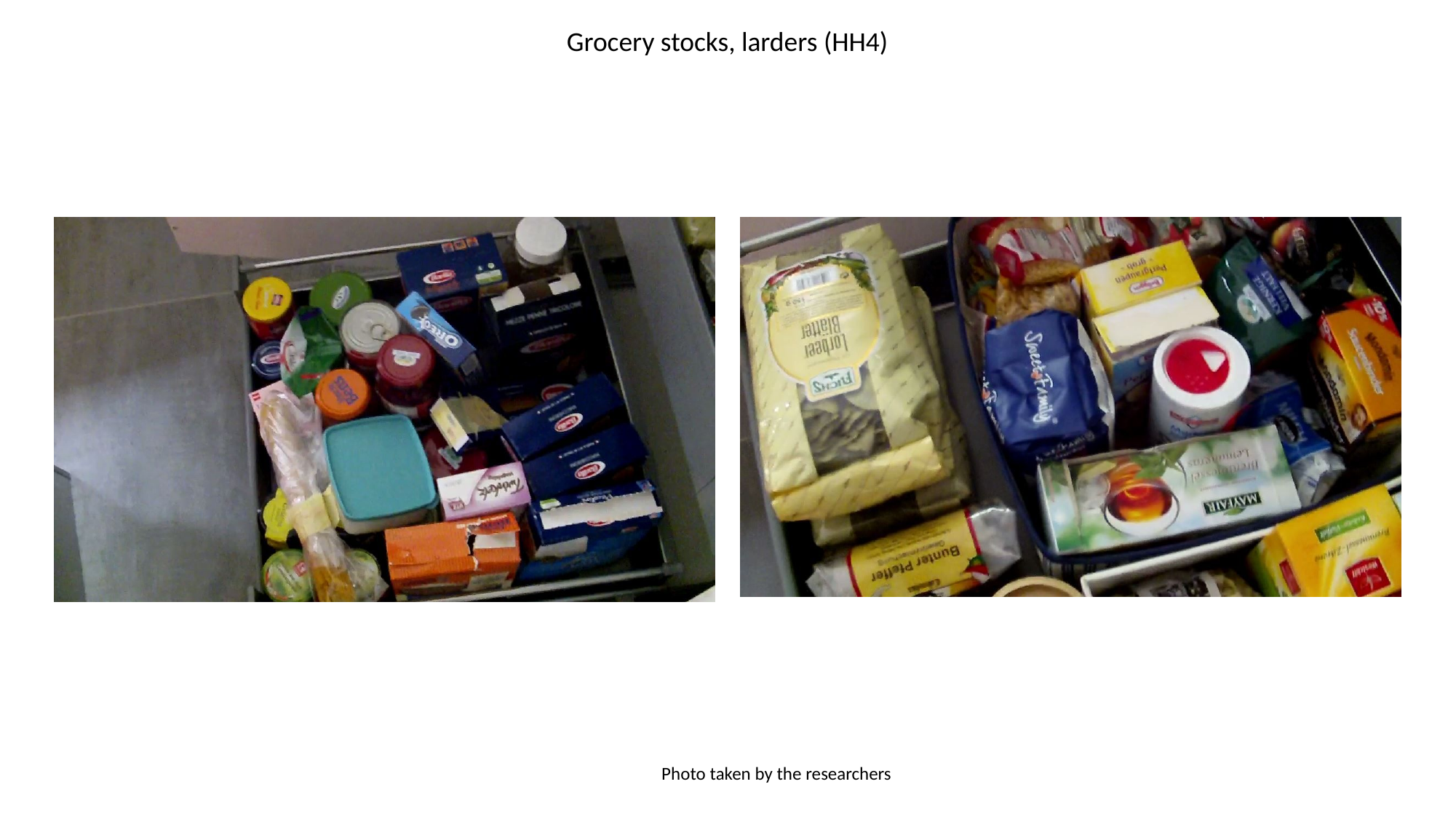

Grocery stocks, larders (HH4)
Photo taken by the researchers

## Slide 14
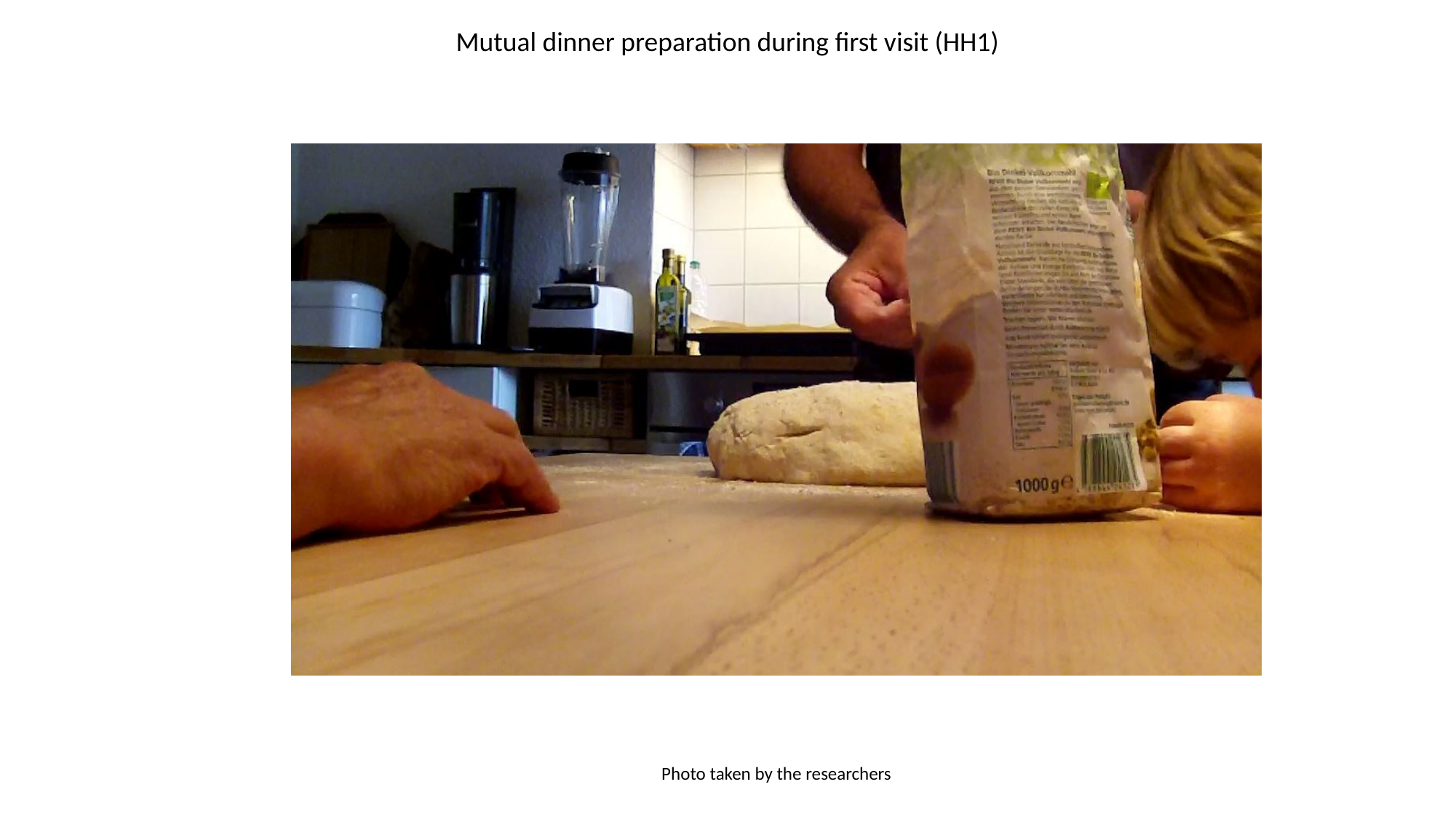

Mutual dinner preparation during first visit (HH1)
Photo taken by the researchers

## Slide 15
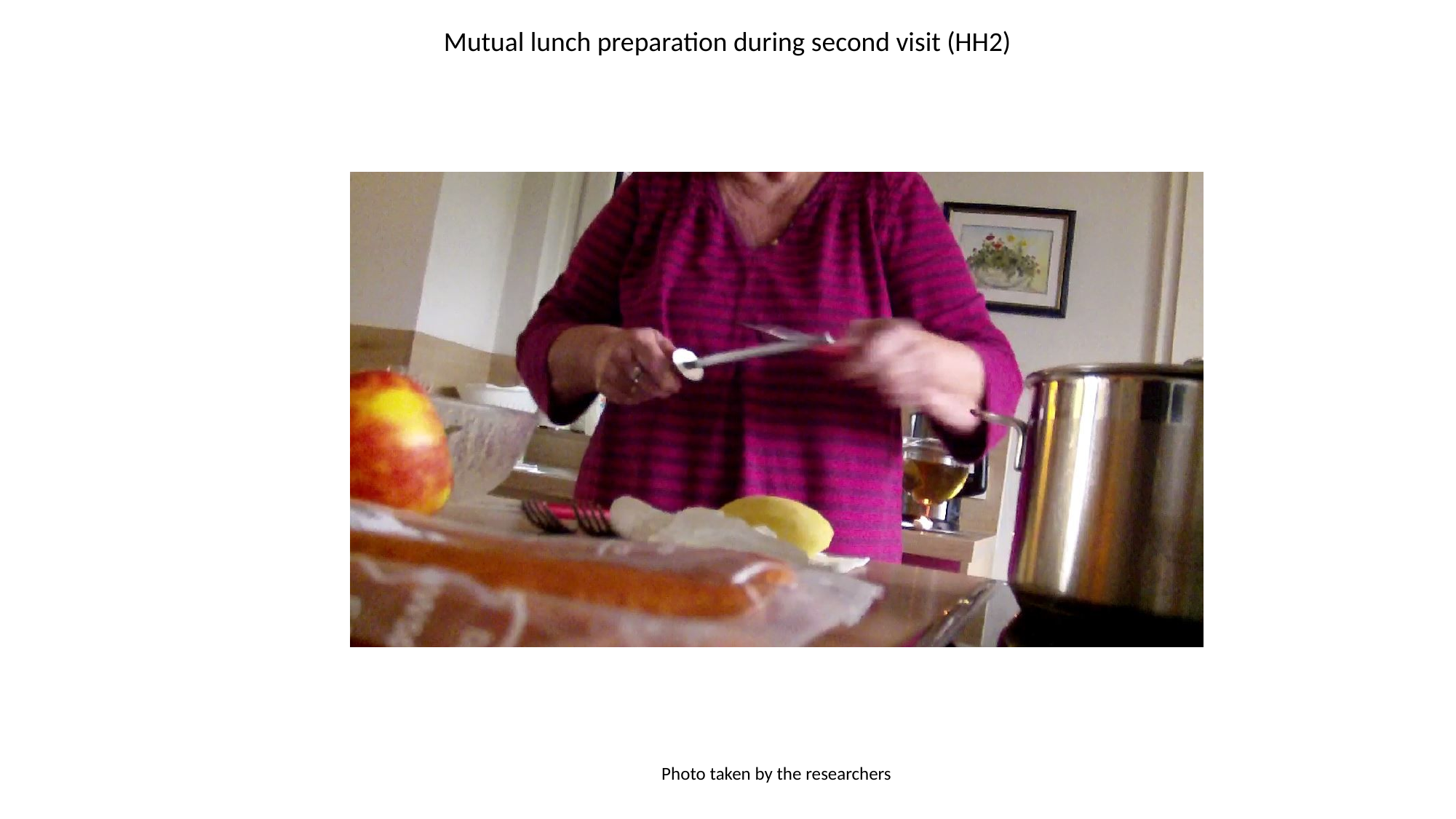

Mutual lunch preparation during second visit (HH2)
Photo taken by the researchers

## Slide 16
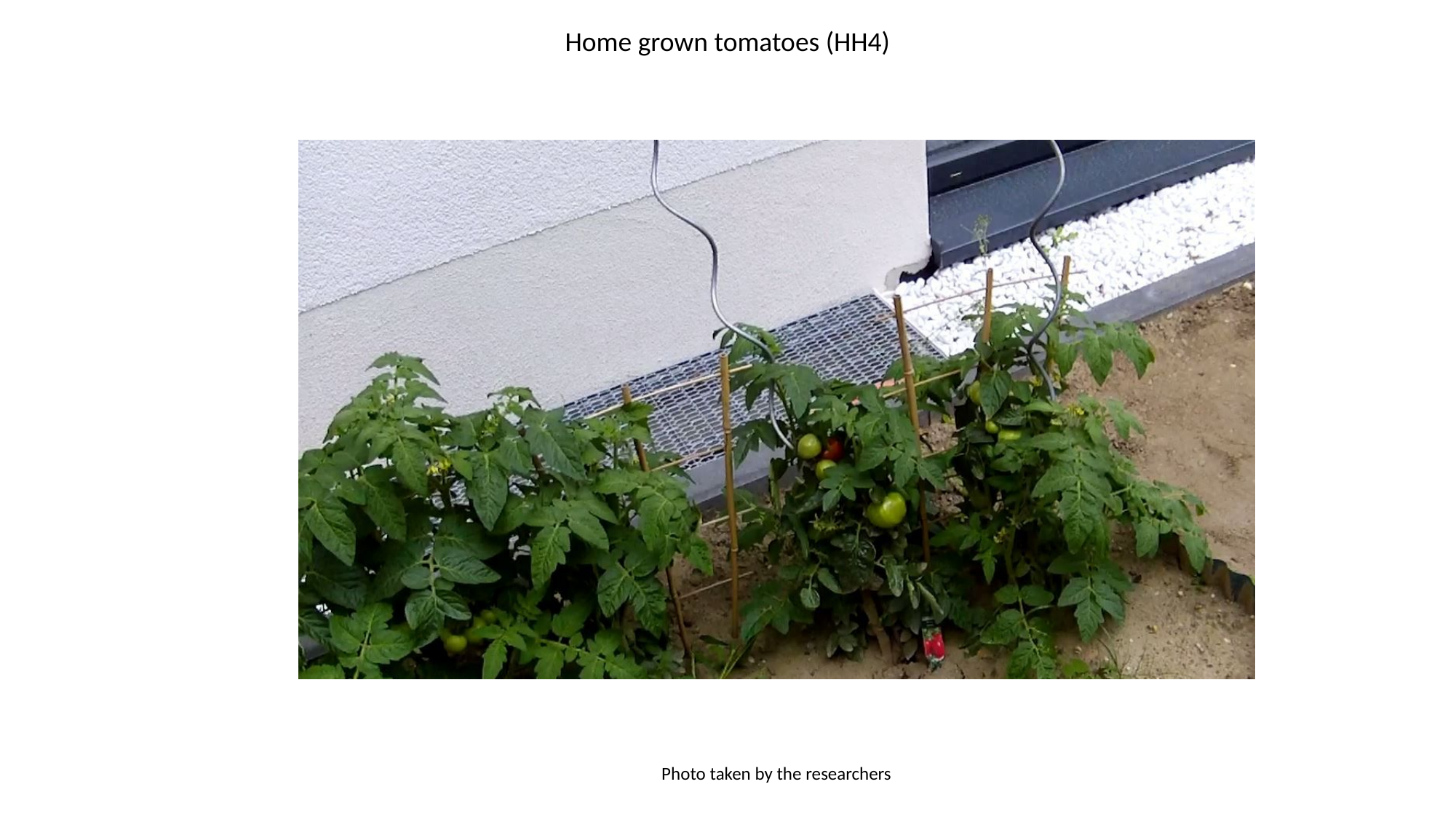

Home grown tomatoes (HH4)
Photo taken by the researchers
