# Supplementary material for: Good Attitudes Are Not Good Enough: An Ethnographical Approach to Investigate Attitude-Behavior Inconsistencies in Sustainable Choice
Source: Foods. 2021 Jun 8;10(6):1317. doi: 10.3390/foods10061317 (PMC8226450; doi:10.3390/foods10061317)
Supplement: Supplementary file 1 [file foods-10-01317-s001.zip › SB_Codebook.pdf]

Supplementary material A

Code	Development	Description	Main Theme
**1 Basic Codes**	Inductive codes, developed during initial coding		
**1.1 Food Perceptions and Behavior**	Inductive, developed during initial coding	Food perception and food behavior	
Bad Worse	Inductive, developed during initial coding	Evaluate/categorize food or food behavior as “bad” or worse compared to other food or food behavior	
Conflict	Inductive, developed during initial coding	Food or food behavior conflicts. E.g. within own behavior, with family members, with time management, with other food attributes etc.	
DIY	Inductive, developed during initial coding	Homemade dishes, home grown food etc.	Cue: self-made dishes are associated with health benefits
Good Better	Inductive, developed during initial coding.	Evaluate/categorize food or food behavior as “good” or better compared to other food or food behavior	
Habit	Inductive, developed during initial coding	Consumption habits and patterns. How they "normally" or "regularly" do things related to food and food behavior	
Inconsistency	Inductive, developed during initial coding	Inconsistencies and uncertainties in food choice and behavior	
Informal Information Narratives	Inductive, developed during initial coding	Unofficial information e.g. narratives from friends, news articles, social media, documentaries etc.	Information about food comes from unofficial sources and narratives
Opinion	Inductive, developed during initial coding	Strong opinions concerning food and food behavior	
Responsibility	Inductive, developed during initial coding	Self- or consumers responsibility with respect to sustainable food behavior	Consumers feel responsible for sustainable food systems
Short cuts	Inductive, developed during initial coding	Mental short cuts for food evaluations and food choice behavior	
**1.2 Thematic Categories**	Inductive and deductive, developed	Food categories and themes	

	during initial coding		
Dairy Eggs	Inductive, developed during initial coding	Food category: diary and eggs.	
Fair Trade	Inductive, developed during initial coding	Food category: fair trade products	Cue: fair trade products are associated with benefits for farmers and the environment
Fruits Vegetables	Inductive, developed during initial coding	Food category: fruits and vegetables	
Ingredients	Inductive, developed during initial coding	Food theme: ingredients	Cue: the absence/ presence of certain food ingredients is associated with health benefits
Label	Deductive, developed during initial coding	Food theme: food labels	
Organic Label	Deductive, developed during initial coding	Food theme: organic labels (e.g. EU-organic, Demeter, organic retailer label etc.) or labeled products	German organic label is better known as EU organic. In general, there is a positive attitude towards organic labels. Organic Labels from farmer associations are perceived as being stricter and therefor "better"
PGI_PDO	Deductive, developed during initial coding.	Food theme: PDO/PGI label or labeled products	PGI_PDO Label is not known, but appreciated
Private Label	Deductive, developed during initial coding	Food theme: private labels or labeled products	Private and retailer label are well known and trusted.
Regional Label	Deductive, developed during initial coding	Food theme: labels indicating the region of origin or about labeled products	Label indicating the region of origin are less known, but appreciated
Meat	Inductive, developed during initial coding	Food category: meat and meat products	Cue: meat consumption is associated with health risks.<br>Meat consumption in general is perceived as bad.
Media	Inductive, developed during initial coding	Food theme: media coverage and information from media incl. social media	Media information is heavily relied on, but contradicting.
Organic	Deductive, developed during initial coding	Food theme: organic food in general. (Also includes every phrase coded	Cue: organic food is associated with health and environmental benefits and

		under the "Organic Label")	with higher animal welfare standards
Pesticides and Antibiotics	Inductive, developed during initial coding	Food theme: pesticides and antibiotics	Cue: the absence of antibiotics in animal breeding and antibiotics in plant cultivation are associated with health and environmental benefits and with a better animal welfare
Price	Inductive, developed during initial coding	Food theme: food prices	
Processed Food	Inductive, developed during initial coding	Food theme: processed food products and ready to eat meals	Cue: consuming highly processed food is associated with health risks
Public Procurement	Deductive, developed during initial coding	Food theme: public procurement e.g. school meals, canteens	
Region of Origin & Country of Origin_	Inductive, developed during initial coding	Food theme: local and national food in general	Cue: local food is associated with environmental and with social benefits
Seasonal	Inductive, developed during initial coding	Food theme: seasonal food in general	Cue: consuming seasonal food is associated with social benefits and environmental benefits
Supermarket	Inductive, developed during initial coding	Food theme: supermarkets	
Conventional	Inductive, developed during initial coding	Food theme: conventional supermarkets	Conventional supermarkets and discounters are main grocery suppliers.
Organic	Inductive, developed during initial coding	Food theme: organic supermarkets	Shopping at an organic market is associated with health benefits, with environmental benefits and provides a good feeling
Specialty and Farmer Market	Inductive, developed during initial coding	Food theme: specialty markets and farmer markets	Shopping at a farmer's market or food form small scale producers is associated with social benefits, health benefits, environmental benefits and provides a good feeling
**2 Life Course and Influences FCPM**	Inductive and deductive, developed during initial coding and recoding	Food choice development over the life course and influences	
Life Course	Deductive, developed during initial coding and recoding	Life course developments and experiences and its influence on food choice behavior	The life course forms food trajectories. Those affect the awareness and importance one attaches to suitability

			of food-related issues as an adult. It can foster or impede sustainable food choice behavior
Food Context Point of Sale	Deductive, developed during initial coding and recoding	Influence of food context and especially point of sale on food choice behavior	The food context within the immediate neighborhood contributes to the perceived "lack of availability" of sustainable food products. Due to a low distribution of appropriate retailers in the immediate neighbourhood participants shop at conventional supermarkets and discounters. The food context can impede sustainable food choice behavior
Ideals	Deductive, developed during initial coding and recoding	Influence of personal ideals on food choice behavior	A positive attitude towards sustainable food products and behavioural intentions are strongly influenced by ideals and societal expectations. Ideals can foster sustainable food choice behavior
Personal Factors	Deductive, developed during initial coding and recoding	Influence of personal factors like food preferences and boundaries and roles on food choice behavior	Personal food boundaries and internalized roles are hard to overcome. They can impede or foster sustainable food choice behavior
Resources	Deductive, developed during initial coding and recoding		Sufficient resources are necessary to successfully implement a sustainable diet
Equipment	Deductive, developed during initial coding and recoding	Influence of material resources e.g. cooking equipment on food choice behavior	An adequate cooking equipment can foster sustainable food choice behavior
Financial	Deductive, developed during initial coding and recoding	Influence of financial resources and financial status on food choice behavior	Sufficient financial resources are a precondition for sustainable food choice behavior
Knowledge	Deductive, developed during initial coding and recoding	Influence of knowledge of sustainability and food on food choice behavior	A lack of knowledge or the inability to determine the reliability and usefulness of information can impede sustainable

			food choice behavior
Skills	Deductive, developed during initial coding and recoding	Influence of skills on food choice behavior	Cooking skills can foster sustainable food choice behavior
Time	Inductive, developed during initial coding and recoding	Influence of the availability of time on food choice behavior	A lack of time can impede sustainable food choice behavior
Social Factors	Deductive, developed during initial coding and recoding	Influence of social Factors on food choice behavior	The social system and relationships the household is embedded in can foster or impede sustainable food choice behavior
**3 Food Values FCPM**	Inductive and deductive, developed during initial coding and recoding	Contrasting diverse food values	
Animal Welfare	Inductive, developed during initial coding and recoding	Animal welfare and animal health in food production	Cue: organic food is associated with higher animal welfare standards. The value is a driver for attitudes and behavioral intentions.
Convenience	Deductive and inductive, developed during initial coding and recoding. Includes convenience of product preparation (deductive) and convenience of product shopping (inductive)	Convenience of food preparation and food shopping	Convenience while shopping for products is more important than convenience while preparing food. The value can compete with sustainability values
Cost	Deductive, developed during initial coding and recoding	Cost of food products	Though prices of a single sustainable product are rejected as competing value, participants admit that exclusively shopping for sustainable products would be too expensive. The value can compete with sustainability values
Environmental Protection	Inductive, developed during initial coding and recoding.	Environmentally friendly food production.	Environmental protection is a driver for attitudes and behavioral intentions
Cultivation	Inductive, developed during recoding	Environmental protection though cultivation methods	Cue: organic production methods and small-scale retailers/ producers are associated with eco-friendly cultivation methods
Packaging Waste	Inductive, developed during recoding	Environmental protection though	Cue: plastic packaging is perceived as

		avoidance of packaging and especially plastic waste	being harmful for the environment
Transport	Inductive, developed during recoding	Environmental protection though short transport distances	Cue: long transport distances are perceived as being harmful for the environment
Waste	Inductive, developed during recoding	Environmental protection though the avoidance food waste	Cue: food waste is perceived as being harmful for the environment
Health	Deductive, developed during initial coding and recoding	Healthiness of food products	Cue: organic food, unprocessed food, unpacked food, food from small scale producers /retailers, the absent of certain ingredients and a diet without certain products is associated with health benefits. The value is a driver for attitudes and behavioral intentions
Managing Relationship	Deductive, developed during initial coding and recoding	Relationships issues, balancing varying preferences, keeping the harmony	Satisfying the needs of all family members and balancing varying preferences is crucial for a harmonic family life. The value can compete with sustainability values
Sensory Perception	Deductive, developed during initial coding and recoding		Sensory perceptions can compete with sustainability values
Other	Deductive, developed during initial coding and recoding	Sensory perceptions like appearance and shelf life of food products	Shelf live, smell and appearance can compete with sustainability values
Taste	Deductive, developed during initial coding and recoding	Taste of food products	Taste is an exclusionary value. If products are not tasty, they are nor bought. Other values cannot compensate the value taste
Social Responsibility	Deductive, developed during initial coding and recoding	Social responsibilities for local community/famers/shop owners in food choice behavior	Local food, food from small scale producers and retailers like farmer markets and specialty shops as well as fair trade products are associated with social benefits. The value is a driver for attitudes and behavioral intentions
**4 Strategies FCPM**	Deductive, developed during initial coding and recoding	Food Choice Strategies	

Assortment	Inductive, developed during initial coding	Assortment strategies	Using the retailer's assortment and product arrangement to facilitate (sustainable) food choice
Passing by	Inductive, developed during initial coding	Passing by strategy in food choice	Visiting those retailers that are close to their place of residence or on their way to/from work/school to facilitate food choice
Choice Architecture	Inductive, developed recoding	Role of the choice architecture in food choice	Changing the default choice architecture can facilitate (sustainable) food choice.
Price	Inductive, developed during initial coding and recoding	Price strategy in food choice	Using food prices to facilitate food choice and food evaluations at supermarket
Avoid Limit Substitute	Inductive, developed during initial coding	Avoidance, limitation and substitution	Participants avoid, limit and substitute certain food products to facilitate (sustainable) choice
Satisficing	Inductive, developed during recoding	Opting for a satisfactory product	Products must not be optimal in terms of perceived sustainability, satisfaction is sufficient.
Simplifications	Inductive, developed during initial coding and recoding.	Simplification and Mental Short Cuts	Usage of simplifications and short cuts to facilitate food evaluations and choice
Product Category Restriction	Inductive, developed during initial coding and recoding	Category restrictions	Restricting sustainable choice to certain product categories
**5 Reasons for ABG**	Inductive, developed during recoding.	Reasons for the Attitude-Behavior Gap	
Interfering Influences	Inductive, developed during initial coding and recoding	Influences interfere with sustainable food choice behavior	
Lack of availability	Inductive, developed during initial coding and recoding	A lack of availability interferes with sustainable food choice behavior	A lack of availability of sustainable food products at retailers and a lack of retailers that offer sustainable food in the immediate neighborhood
Lack of time	Inductive, developed during initial coding and recoding	A lack of time interferes with sustainable food choice behavior	A lack or shortage of time hinders participants to visit a retailer that offers sustainable food products or prepare meals they perceive as sustainable

Relationship	Inductive, developed during initial coding and recoding	Personal relationships interfere with sustainable food choice behavior	Conflicting preferences of family members/ peers can prevent participants to buy the sustainable product. Other family members do not attach the same importance to sustainability issues
Tradition Habit	Inductive, developed during initial coding and recoding	Personal and family member traditions and habits interfere with sustainable food choice behavior	Traditions and habits have strong behavioral influence which can hinder sustainable food choice behavior
Competing other food values	Inductive, developed during initial coding and recoding	Competing food values interfere with sustainable food choice behavior	
Div. Disadvantages	Inductive, developed during initial coding and recoding	Disadvantage of sustainable products	Diverse disadvantages of sustainable food products compared to other food products e.g.: different appearance, a shorter shelf life, higher efforts of preparations etc. can hinder sustainable food choice behavior
Price	Inductive, developed during initial coding and recoding	Higher prices for sustainable products	Comparatively higher prices of sustainable products can hinder sustainable food choice behavior
Taste	Inductive, developed during initial coding and recoding	Taste of sustainable food products	A different /worse / not liked taste of the sustainable product can hinder sustainable food choice behavior
Conflicting sustainability values	Inductive, developed during initial coding and recoding	Conflicting sustainability values interfere with sustainable food choice behavior	Sustainability values conflicting with each other e.g.: organic vs. packed, organic vs. local etc. lead to uncertainties and frustration
Lack of useful information Uncertainties	Inductive, developed during initial coding and recoding	A lack of useful information and uncertainties interfere with sustainable food choice behavior	A lack of useful information and uncertainties concerning sustainability issues and sustainable food products hinders sustainable food choice
**6. Dealing with inconsistencies**	Inductive, developed during initial coding and recoding	Covers how participants deal/make sense of inconsistencies	

Neutralization/Justification	Inductive, developed during initial coding and recoding	Covers neutralizations and justifications	Neutralization or justification of unsustainable choice
Additional effort	Inductive, developed during initial coding and recoding	Additional effort	Justifying unsustainable food choice with "too much additional effort"
Exceptions	Inductive, developed during initial coding and recoding	Exception and Excuses	Justifying unsustainable food choice with that the unsustainable choice is an exception
Acceptance	Inductive, developed during initial coding and recoding	Acceptance	Acceptance of unsustainable behavior.
No Recognition	Inductive, developed during initial coding and recoding	Not recognizing unsustainable choice	Unsustainable choice are not recognized
